# Characterisation of the periodontal proteome in gingival crevicular fluid and saliva using SWATH-MS

**DOI:** 10.3389/fcimb.2025.1576906

**Published:** 2025-05-02

**Authors:** Triana Blanco-Pintos, Alba Regueira-Iglesias, Berta Suárez-Rodríguez, Noelia Seijas-Otero, Marta Relvas, Susana B. Bravo, Carlos Balsa-Castro, Inmaculada Tomás

**Affiliations:** ^1^ Oral Sciences Research Group, Special Needs Unit, Department of Surgery and Medical-Surgical Specialties, School of Medicine and Dentistry, Universidade de Santiago de Compostela, Health Research Institute of Santiago (IDIS), Santiago de Compostela, Spain; ^2^ Oral Pathology and Rehabilitation Research Unit (UNIPRO), University Institute of Health Sciences (IUCS-CESPU), Gandra, Portugal; ^3^ Proteomic Unit, Health Research Institute of Santiago de Compostela (IDIS), Santiago de Compostela, Spain

**Keywords:** periodontitis, gingival crevicular fluid, saliva, oral fluids, protein expression, SWATH-MS, mass spectrometry, proteomics

## Abstract

**Introduction:**

Proteomic techniques are useful to analyse the periodontal proteome in gingival crevicular fluid (GCF) and saliva. However, few investigations have assessed and compared the GCF and salivary proteomes. Therefore, this research aims to analyse the proteome structure and compare protein expression in these fluids between individuals with periodontal health and those with periodontitis.

**Methods:**

GCF and saliva were collected from 44 periodontally healthy subjects and 41 with periodontitis (stages III-IV). Samples were analysed using sequential window acquisition of all theoretical mass spectra (SWATH-MS), and proteins were identified employing the UniProt database. The periodontal proteome structure was assessed using principal component analysis (PCA). Differential protein expression was defined as an adjusted p-value <0.05 combined with a fold-change ≥2 (upregulated) or ≤0.5 (downregulated).

**Results:**

250 abundant proteins were quantified in GCF and 377 in saliva (238 in common). The proteome structure was different in periodontitis compared to periodontal health in both oral fluids. In GCF, 63 (25.2%) proteins were differentially expressed, with 38 upregulated and 25 downregulated in periodontitis. The most overexpressed proteins were haemoglobin subunits (Hbs) beta (fold-change of 5.06) and alpha (4.35), carbonic anhydrase 1 (4.28), and protein S100-P (4.27). Among the underexpressed proteins, 14 were keratins, with type II cytoskeletal 6B being the most downregulated (0.10), together with glyceraldehyde-3-phosphate dehydrogenase (0.12) and zymogen granule protein 16 homolog B (0.13).

In saliva, 59 (15.7%) proteins were differentially expressed, with 55 upregulated and four downregulated in periodontitis. Twenty-nine proteins showed a fold-change ≥4, highlighting beta-2-microglobulin (44.14), keratin, type I cytoskeletal 13 (36.23), neutrophil defensin 1 (25.08), proteins S100-A9 (12.30), A8 (10.61), A12 (4.76), and P (4.72), annexin A1 (9.34), lysozyme C (4.98), immunoglobulin heavy constant alpha 1 (4.45), resistin (4.37), and Hbs beta (4.20) and alpha (4.06). The most downregulated protein was lipocalin-1 (0.35). Fourteen proteins were differentially expressed in GCF and saliva, where seven were keratins being underexpressed in GCF but overexpressed in saliva.

**Conclusion:**

Periodontitis alters the periodontal proteome structure and the expression of numerous abundant proteins in GCF and saliva. However, proteins expressed vary qualitatively and quantitatively, indicating different expression patterns between oral fluids.

## Introduction

Periodontitis is a chronic inflammatory disease that affects the gingiva, the alveolar bone, and the periodontal ligament surrounding the teeth (Kinane et al., 2017). Periodontitis not only has an impact on oral health but is also related to numerous systemic diseases, being especially relevant in their association with cardiovascular diseases or diabetes (Monsarrat et al., 2016). Today, periodontitis remains one of the most prevalent diseases worldwide, affecting almost 45% of the world’s population (Eke et al., 2020).

Several proteins have been shown to play an essential role in the initiation, progression, and severity of periodontitis (Bostanci and Belibasakis, 2018). However, other relevant proteins have not yet been identified, and their associations with the disease remain undiscovered (Bostanci and Belibasakis, 2018). Analyses using high-throughput proteomic techniques enable the periodontal proteome to be studied in greater detail, contributing to the determination of extensive and comprehensive protein profiles associated with periodontal diseases (Bostanci and Bao, 2017; Bostanci and Belibasakis, 2018).

Considering the range of proteomic techniques available, sequential window acquisition of all theoretical mass spectra (SWATH-MS) stands out as a particularly effective approach (Anjo et al., 2017). SWATH-MS is an untargeted, data-independent acquisition (DIA), label-free quantification technique designed to eliminate the variability introduced by chemical modifications, expand the dynamic range of quantification, and optimise the detection of data present in a sample (Frederick and Ciborowski, 2016). This method was developed to combine the proteome coverage potential of data-dependent acquisition (DDA) approaches with the quantitative consistency of targeted DIA proteomics (e.g., selected reaction monitoring – SRM) (Ludwig et al., 2018). These features make SWATH-MS a powerful strategy for discovering novel biomarkers (Anjo et al., 2017).

The most commonly used sample types for studying the periodontal proteome are gingival crevicular fluid (GCF) and saliva. Both can be easily collected in a non-invasive manner, making them particularly suitable for potential translation into clinical diagnostic tools (Guzman et al., 2014). The analysis of these fluids using various proteomic approaches has enabled the identification and quantification of numerous proteins with significantly different expression profiles depending on periodontal status (Ahmad et al., 2025). However, few studies have simultaneously analysed the GCF and salivary proteome collected from the same individuals (Grant et al., 2022; Tang et al., 2019). Additionally, SWATH-MS has previously proven useful in identifying novel GCF and salivary biomarkers with strong diagnostic potential for periodontitis (Blanco-Pintos et al., 2024, 2025a). Nevertheless, to date, SWATH-MS has not been employed to comprehensively study and compare the proteomic profile and protein expression between periodontal health and periodontitis.

Accordingly, this cross-sectional study aimed to (1) analyse the periodontal proteome structure and (2) compare differential protein expression in GCF and saliva between individuals with periodontal health and those with periodontitis using SWATH-MS.

## Material and methods

**Figure 1:**
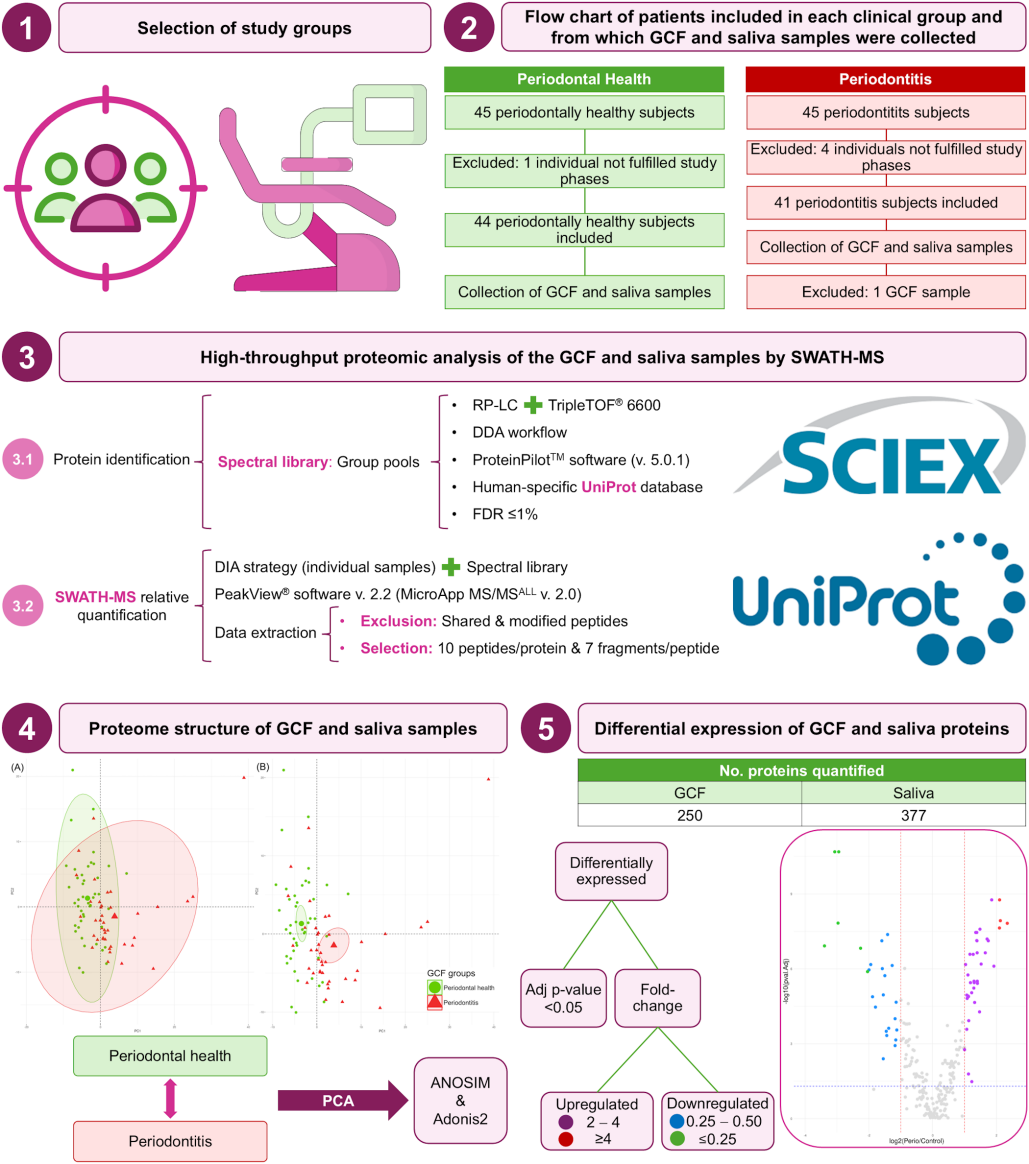
Complete analysis protocol of this study. Adj, adjusted; ANOSIM, analysis of the similarities; DDA, data-dependent acquisition; DIA, data-independent acquisition; FDR, false discovery rate; GCF, gingival crevicular fluid; LC, liquid-chromatography; No., number; PCA, principal component analysis; RP, reversed-phase; SWATH-MS, sequential window acquisition of all theoretical mass spectra; TOF, time-of-flight; v., version.

The complete analysis protocol applied in the present study is detailed in **Figure 1**.

### Selection of study groups

A convenience sample of 90 eligible participants, comprising 45 periodontally healthy subjects (control group) and 45 individuals affected by untreated periodontitis (periodontitis group) was recruited between 2018 and 2021 from 250 consecutive patients in the general population who were referred for assessment of their oral health status to the School of Medicine and Dentistry (Universidade de Santiago de Compostela, Spain) and the Instituto Superior de Ciências da Saúde-Norte, Cooperativa de Ensino Superior, Politécnico e Universitário (CESPU, Gandra, Paredes, Portugal).

Patients were selected if they fulfilled the following inclusion criteria: 1) aged 20 to 75; 2) had at least 10 natural teeth; 3) no previous periodontal treatment; 4) had no medical history of diabetes mellitus, hepatic or renal disease, or other severe medical conditions or transmittable illnesses; 5) had not taken systemic antimicrobials in the previous six months; 6) had no intake of antiinflammatory medication in the previous four months; 7) did not routinely use oral antiseptics; 8) had no history of alcohol or drug abuse; 9) were not pregnant or breastfeeding; and 10) used no orthodontic appliances.

Two experienced and previously calibrated dentists performed the periodontal diagnostics, with intra- and interexaminer intraclass correlation coefficients (ICC) greater than 0.950. Bleeding on probing (BOP) and the bacterial plaque level (BPL) were recorded for the entire mouth on a binary scale (presence/absence) at six sites per tooth. The probing pocket depth (PPD) and clinical attachment loss (CAL) were recorded using a PCP-UNC 15 probe at six sites per tooth. PPD was measured as the distance from the gingival margin to the base of the gingival sulcus. A positive value for gingival recession (REC) was assessed for the apical displacement of the gingival margin in relation to the cementoenamel junction. Considering an average biological width (BW) of 2 mm (Mulla et al., 2023), CAL was defined for three conditions: (1) PPD <4 mm and REC of 0 mm, CAL was equal to 0 mm; (2) PPD ≥4 mm and REC of 0 mm, CAL was equal to PPD subtracting BW; and (3) if REC >0 mm, CAL was PPD plus REC.

Standardised teeth radiographs were acquired to assess the alveolar bone status. The diagnosis of periodontitis was based on the clinical and radiographic information thus obtained. The control group included subjects who were periodontally healthy according to the criteria in the Classification of Periodontal Diseases and Conditions (Caton et al., 2018; Tonetti et al., 2018), i.e., BOP <10%, no location with a PPD ≥4 mm, and no radiographic evidence of alveolar bone loss. This classification was also used for the periodontitis group, which was composed of patients diagnosed with stage III or IV and grades B or C generalised periodontitis.

A questionnaire was used to evaluate the smoking habits of the participants, collecting information on their smoking status (non-smoker, former smoker, current smoker), the duration of their smoking status (as a former or current smoker), and the number of cigarettes consumed per day. Non-smokers were considered those who had never smoked, current smokers were those who had been smoking for at least one year, and former smokers were those who stopped doing so at least one year before, irrespective of how long they had been smoking.

The research was conducted following the principles of the Declaration of Helsinki (revised in 2013) on human experimentation studies (World Medical Association, 2013). The protocol was approved by the Galician Clinical Research Ethics Committee (registration numbers 2018/295 and 2021/417) and the Instituto Superior de Ciências da Saúde-Norte, CESPU (registration number 35/CEIUCS/2019). All the participants provided written informed consent to their involvement in the study.

### Collection of the GCF and saliva samples and preparation for the mass spectrometry analysis

The GCF and saliva samples were collected one or two weeks after the initial examination. The GCF samples from the control and periodontal subjects were collected and pooled from five non-adjacent proximal sites. The samples were taken from teeth in quadrants 1 and 3 in the periodontally healthy controls. In patients with periodontitis, samples were collected from the sites with the deepest PPD values. Two paper strips were inserted into the gingival sulcus or periodontal pocket for 30 seconds. In cases of visible contamination with blood, samples were discarded, and new sites were sampled. The strips were collected and inserted into labelled tubes containing 300 mL of 0.01 M phosphate-buffered saline (PBS) with a pH of 7.2.

Immediately before the collection of the GCF samples, one ml of unstimulated whole saliva was collected from each participant using the spitting method at least one hour after the last meal and after the last brushing (Navazesh and Christensen, 1982). Subsequently, the GCF and saliva samples were stored at -80°C until further analysis.

The total protein concentrations of the samples were quantified using the reducing-agent and detergent-compatible (RC DC™) Protein Assay Kit (BIO-RAD, Hercules, CA, USA), following the manufacturer’s protocol. Approximately 50 μg of protein from each sample was precipitated using the methanol/chloroform (MeOH/CHCl_3_) method to remove potential contaminants such as deoxyribonucleic acid (DNA), ribonucleic acid (RNA), and lipids. Purified GCF and salivary proteins were loaded onto a 10% sodium dodecyl sulfate-polyacrylamide gel electrophoresis (SDS-PAGE) gel to concentrate them into a band for in-gel digestion. Electrophoresis was interrupted once the front had penetrated 3 mm into the gel (Bonzon-Kulichenko et al., 2011; Perez-Hernandez et al., 2013). The protein band was detected with SYPRO Ruby fluorescent staining (Lonza, Basel, Switzerland) and cut out. We then proceeded to manual in-gel tryptic digestion, as previously explained (Shevchenko et al., 1996), but with slight modifications. The gel pieces were reduced with 10 mM dithiothreitol (Sigma-Aldrich, St. Louis, Missouri, USA) in 50 mM ammonium bicarbonate (Sigma-Aldrich) and alkylated with 55 mM iodoacetamide (Sigma-Aldrich) in 50 mM ammonium bicarbonate.

The gel pieces were then rinsed with 50 mM ammonium bicarbonate in 50% MeOH (HPLC grade, Scharlau, Barcelona, Spain), dehydrated by adding acetonitrile (ACN) (HPLC grade, Scharlau), and dried in a SpeedVac (Savant™). Modified porcine trypsin (Promega, Madison, Wisconsin, USA) was added to the dried gel pieces at a final concentration of 20 ng/μL in 20 mM ammonium bicarbonate and incubated at 37°C for 16 hours. Peptides were extracted three times by way of a 20-minute incubation in 40 μl of 60% ACN in 0.5% acetic acid (HCOOH). The resulting peptide extracts were pooled, concentrated in a SpeedVac, and stored at -20°C until use.

### Protein quantification by SWATH-MS

#### Construction of MS/MS spectral libraries

To obtain a robust representation of the peptides and MS/MS present in the GCF and saliva study groups, four pools of around 10 samples from each group (periodontal health and periodontitis) were prepared after protein digestion using equal amounts of peptides from each sample. In more detail, after peptide resuspension in water plus 0.1% of formic acid with sonication per 10 minutes (the resuspension was made to obtain a µg/µl peptides solution), from each of the resuspended samples/peptides, we used three µl for create the corresponding pool to obtain the spectral library (Blanco-Pintos et al., 2024, 2025a, 2025b; Bugallo-Casal et al., 2025; Casado-Fernández et al., 2024; López-Valverde et al., 2024; Mondelo-Macía et al., 2024; Nóvoa et al., 2025; Rodrigues et al., 2024; Torres Iglesias et al., 2024; Vázquez-Mera et al., 2024). Four µl (around four µg) of each pool were separated using reversed-phase (RP) LC to identify the proteins and create the SWATH library.

The gradient was created with a micro-LC system (Eksigent Technologies nanoLC 400, SCIEX, Redwood City, California, USA) coupled with a TripleTOF^®^ (time-of-flight) 6600 high-speed mass spectrometer (SCIEX) via a microflow ionisation source. A silica-based RP analytical column (C18CL 150×0.30 mm) with a particle size of 3 µm and a pore size of 120 Å (Eksigent, SCIEX) was employed. The samples were pre-washed on a YMC-TRIART C18 pre-column (YMC Technologies, Teknokroma, Barcelona, Spain), which had the same particle and pore sizes, and was connected in-line with the analytical column. The charge pump provided a 0.1% formic acid solution in water at a rate of 10 μl/min. The micropump generated a flow rate of 5 μl/min and operated under gradient elution conditions, employing 0.1% formic acid in water as mobile phase A and 0.1% formic acid in ACN as mobile phase B. Peptides were separated using a 40-minute gradient from 2% to 90% of mobile phase B. The injection volume was 4 μl (approximately 4 μg of protein).

The TripleTOF 6600 (SCIEX) was used for data acquisition, employing a DDA workflow. The source and interface conditions comprised an ion-source voltage (ISVF) of 5500V, a curtain gas (CUR) of 25, a collision energy (CE) of 10, and an ion-source gas 1 (GS1) of 25. The instrument was operated using the software Analyst TF 1.7.1 (SCIEX). Switching criteria were set for ions with a mass-to-charge ratio (*m/z*) ranging from 350 to 1400, a charge state of 2-5, a mass tolerance of 250 parts per million (ppm), and an abundance threshold of more than 200 counts per second (cps). Selected ions meeting these conditions were excluded for 15 seconds. The instrument was automatically calibrated every four hours using commercial PepCalMix tryptic peptides (SCIEX) as the external calibrants.

After the MS/MS analysis, the data files were processed using ProteinPilotTM 5.0.1 (SCIEX), which employs the ParagonTM algorithm for database search and Progroup™ for data grouping. Files were searched for peptide/protein identification against the UniProt human-specific database (UniProt Consortium, 2014) (https://www.uniprot.org/, UniProt release 2022_02, containing 20387 human proteins), specifying iodoacetamide at cysteine alkylation as a variable modification and methionine oxidation as a fixed modification. All known contaminants (keratins epidermal, trypsin…) were manually excluded. The false discovery rate (FDR) was set at ≤1% for both peptides and proteins (Aggarwal and Yadav, 2016). The MS/MS spectra of the identified peptides were used to generate a spectral library, which subsequently enabled the extraction of SWATH peaks using MicroApp MS/MSALL 2.0 for PeakView 2.2 (SCIEX). Only peptides with a confidence score above 99% (obtained by searching against the UniProt database) were included in the spectral library.

#### Relative quantification

The GCF and saliva samples were analysed individually, using a DIA method. These were then examined using the equipment and gradients described above for spectral library construction, although a SWATH-MS acquisition strategy was employed instead of a DDA approach. The method involves repeating an acquisition cycle. In particular, 100 TOF MS/MS scans (400 to 1500 *m/z*, high sensitivity mode, and 50 millisecond acquisition time) were performed using sequential, overlapping precursor isolation windows of varying widths (1 *m/z* overlap) that cover the entire mass range between 400 and 1250 *m/z*, obtained with a previous TOF MS scan (400–1500 *m/z* and 50 millisecond acquisition time) for each cycle. The total cycle time was 6.3 seconds. A SWATH variable window spreadsheet (SCIEX) was used to optimise the width of the 100 variable windows for each set of samples according to the ion density found in the DDA runs.

The extraction of specific data from the trace-ion chromatograms and fragments obtained using the SWATH-MS methodology was performed with the PeakView 2.2 software (SCIEX), utilising MicroApp MS/MS^ALL^ 2.0 and the previously created spectral library. Only peak area values integrated based on signal intensity from 10 peptides per protein and 7 fragments per peptide were selected. Shared and modified peptides were excluded from the processing. Five-minute windows and widths of 30 ppm were used to extract the ion chromatograms. SWATH-MS quantification was performed for all the proteins in the ion library identified by ProteinPilot™ with an FDR ≤1%. The retention times (RTs) of the selected peptides for each protein were realigned in each run according to the peptide-indexed RTs obtained in each sample throughout the chromatogram. The areas of each protein in each sample were acquired by combining the peak areas of the 10 peptides and the seven corresponding fragment ions.

The integrated peak areas (processed PeakView^®^ mrkvw files) were exported directly to the MarkerView™ software (SCIEX) for the relative quantitative examination, which is an approach that has been used previously for SWATH-MS data analyses (Agra-Bermejo et al., 2020; Álvarez et al., 2020; Camino et al., 2020, 2022; Chantada-Vázquez et al., 2020; García-Vence et al., 2020; Novelle et al., 2021; Ortea et al., 2018). This export generated three files containing quantitative information on individual ions, the total intensity of the different ions for a particular peptide, and the total intensity of the different peptides for a specific protein. MarkerView™ utilises processing algorithms that accurately identify chromatographic and spectral peaks directly within the raw SWATH data. The software’s alignment of the data compensates for slight variations in mass and RT values, ensuring precise comparisons of identical compounds in different samples. To homogenise the data obtained, a most likely ratio (MLR) normalisation was performed (Colangelo et al., 2015).

### Statistical analysis

For the calculation of the sample size, we considered the design of independent groups (periodontal health *vs* periodontitis), the possibility of using a nonparametric two-tailed test of mean differences between two independent groups, an effect size of 0.65, an alpha error of 0.05 and a statistical power of 0.80, a minimum sample size of 40 subjects in each group was required. Due to the possible loss of patients for various reasons, each study group initially consisted of 45 participants. Sample size calculation was performed with the G*Power program (version 3.1.9.4) (Faul et al., 2007).

Five of the 90 subjects selected were excluded for non-compliance with the project phases. Therefore, the clinical groups were finally composed of 44 periodontally healthy subjects and 41 patients with periodontitis. One GCF sample from those with periodontitis had to be excluded due to a processing error.

The R software (version 4.2.2) (R Core Team, 2022) was used to perform the statistical data analysis of the GCF and saliva samples from the study groups (control and periodontitis).

#### Comparison of clinical characteristics between control subjects and patients with periodontitis

The Shapiro-Wilk test was used to assess the distribution of quantitative variables. For variables with a normal distribution, the Student’s t-test was applied; otherwise, the Mann-Whitney U test was used. Fisher’s exact test and the Chi-squared test were used to assess the association between qualitative variables and the clinical conditions. A significance level of p<0.05 was established.

#### Periodontal proteome structure

Principal component analysis (PCA) was employed to visualise the clustering of the GCF and saliva samples in each analysis group. The FactoMineR software (version 2.8) (Lê et al., 2008) was used to perform the PCA, and then the amount of variability explained within each component was assessed using the factoextra package (version 1.0.7) (Kassambara and Mundt, 2020). The vegan software (version 2.6.4) (Oksanen et al., 2022) was used to perform two evaluations: 1) an analysis of the similarities (ANOSIM) between groups; and 2) an analysis of variance, using a similarity matrix via the adonis2 function of the non-parametric test permutational multivariate analysis of variance (PERMANOVA) to that end (Anderson, 2001). A significance level of p<0.05 was established.

#### Differential protein expression

Concerning all the proteins quantified in GCF and saliva, we evaluated the mean expression difference between the clinical groups using the non-parametric Mann-Whitney U test. The p-values obtained were adjusted using the mutoss package (version 0.1-13) (Team et al., 2023) with the Benjamini-Hochberg correction (Benjamini and Hochberg, 1995). The differential expression was statistically significant if the adjusted p-value was <0.05 (-log10 adjusted p-value=1.30).

Subsequently, the effect size of each protein in all the phases was calculated using the effsize package (version 0.8.1) (Torchiano, 2020) and Cohen’s d (Cohen, 1988). The expression ratio of each protein between conditions was calculated, and a fold-change ≥2 (log2 fold-change ≥1) or ≤0.5 (log2 fold-change ≤-1) was considered significant.

Volcano plots were created from the adjusted p-values and the differential expression of the log2 fold-change. Proteins with an adjusted p-value <0.05 and a log2 fold-change ≥1 (upregulated) or ≤-1 (downregulated) were differentially expressed.

## Results

### Clinical characteristics

Considering the clinical parameters, full-mouth BPL, BOP, PPD, and CAL levels were significantly higher in patients with periodontitis than in control subjects. Similarly, BOP, PPD and CAL levels from sampled GCF sites were significantly higher in periodontal patients (**Table 1**).

**Table 1:** Age, gender, smoking habit, and clinical characteristics of GCF and saliva sampled patients associated with periodontal status in the control group and periodontitis.

Clinical characteristics	Study groups		
	Control group (n=44)	Periodontitis group (n=41)*	p-value
Age, years	31.86 ± 10.26	53.80 ± 10.09	<0.001^†^
No. male (%)	17 (38.64)	21 (51.22)	NS
No. female (%)	27 (61.36)	20 (48.78)	
Smoking dimensions			
No. patients non-smokers (%)	27 (61.36)	13 (31.71)	0.004
No. patients smokers (%)	15 (34.09)	17 (41.46)	
No. patients ex-smokers (%)	2 (4.55)	11 (26.83)	
No. cigarettes/day	11.26 ± 7.49	16.71 ± 10.29	NS^†^
No. patients <10 cigarettes/day (%)	7 (15.91)	7 (17.07)	NS
No. patients 10–20 cigarettes/day (%)	8 (18.18)	15 (36.59)	
No. patients >20 cigarettes/day (%)	2 (4.55)	6 (14.63)	
No. years of smoking	11.75 ± 6.67	28.39 ± 10.95	<0.001
No. patients <5 years smoking (%)	2 (4.55)	1 (2.44)	NS
No. patients ≥5 years smoking (%)	15 (34.09)	27 (65.85)	
No. years without smoking	7.00 ± 2.83	7.27 ± 5.83	NS^†^
No. patients <5 years without smoking (%)	0 (0.00)	4 (9.76)	NS
No. patients ≥5 years without smoking (%)	2 (4.55)	7 (17.07)	
Clinical parameters			
No. of teeth	27.66 ± 1.12	21.41 ± 4.91	<0.001^†^
No. sites evaluated	165.95 ± 6.72	131.12 ± 29.04	<0.001^†^
BPL, %	14.23 ± 11.03	51.10 ± 27.15	<0.001^†^
No. patients BPL ≤30% (%)	39 (88.64)	8 (19.51)	<0.001
No. patients BPL >30% (%)	5 (11.36)	33 (80.49)	
BOP, %	3.98 ± 2.65	50.95 ± 19.18	<0.001^†^
No. patients BOP <10% (%)	44 (100)	0 (0.00)	<0.001
No. patients BOP 10-30% (%)	0 (0.00)	6 (14.63)	
No. patients BOP >30% (%)	0 (0.00)	35 (85.37)	
PPD, mm	1.75 ± 0.29	3.59 ± 0.58	<0.001
% sites PPD ≤3 mm	100 ± 0.00	55.48 ± 17.49	<0.001^†^
% sites PPD 4–5 mm	0.00 ± 0.00	33.21 ± 10.62	<0.001^†^
% sites PPD ≥6 mm	0.00 ± 0.00	11.31 ± 9.21	<0.001^†^
CAL, mm	0.01 ± 0.03^‡^	3.34 ± 1.80	<0.001^†^
% sites CAL 0 mm	99.77 ± 1.00	31.02 ± 20.74	<0.001^†^
% sites CAL 1–2 mm	0.23 ± 1.00^‡^	12.64 ± 7.99	<0.001^†^
% sites CAL 3–4 mm	0.00 ± 0.00	22.03 ± 8.28	<0.001^†^
% sites CAL ≥5 mm	0.00 ± 0.00	34.31 ± 25.62	<0.001^†^
Sampled sites			
BOP, %	0.85 ± 3.19	87.25 ± 16.48	<0.001^†^
PPD, mm	1.91 ± 0.40	6.09 ± 0.75	<0.001^†^
CAL, mm	0.00 ± 0.00	6.08 ± 1.75	<0.001^†^
Periodontitis stage and grade			
No. patients stage III (%)	NA	29 (70.73)	NA
No. patients stage IV (%)	NA	12 (29.27)	
No. patients grade B (%)	NA	17 (41.46)	NA
No. patients grade C (%)	NA	24 (58.54)	

No., number; BPL, bacterial plaque level; BOP, bleeding on probing; PPD, probing pocket depth; mm, millimetre; CAL, clinical attachment loss; NA, not applicable; NS, not significant.

*One GCF sample from the periodontitis group had to be excluded due to a processing error resulting in 40 patients.

^†^Mann-Whitney U test (non-normal distribution).

^‡^CAL values different from 0 for traumatic reasons, not of periodontal origin.

Values are presented as means ± standard deviations, and in some cases, the number of subjects or the percentage of sites is also provided. A significant p-value of <0.05 was established.

### Proteins identified and quantified in GCF and saliva samples by SWATH-MS

The *in silico* spectral library contained 37985 transitions, 4556 peptides, and 427 proteins. Of these, 250 GCF and 377 salivary proteins were quantified by SWATH-MS using the previously defined criteria. Interestingly, 238 proteins were quantified in both oral fluids, representing 95.2% of the GCF proteins and 63.1% of those in saliva (**Appendix S1**).

### Periodontal proteome structure in GCF and saliva

The periodontal proteome structure of the GCF and salivary proteins was evaluated using a PCA. The first two principal components or dimensions accounted for 37.3% of the total variance in GCF and 23.0% in saliva (**Appendix S2**).

**Figure 2:**
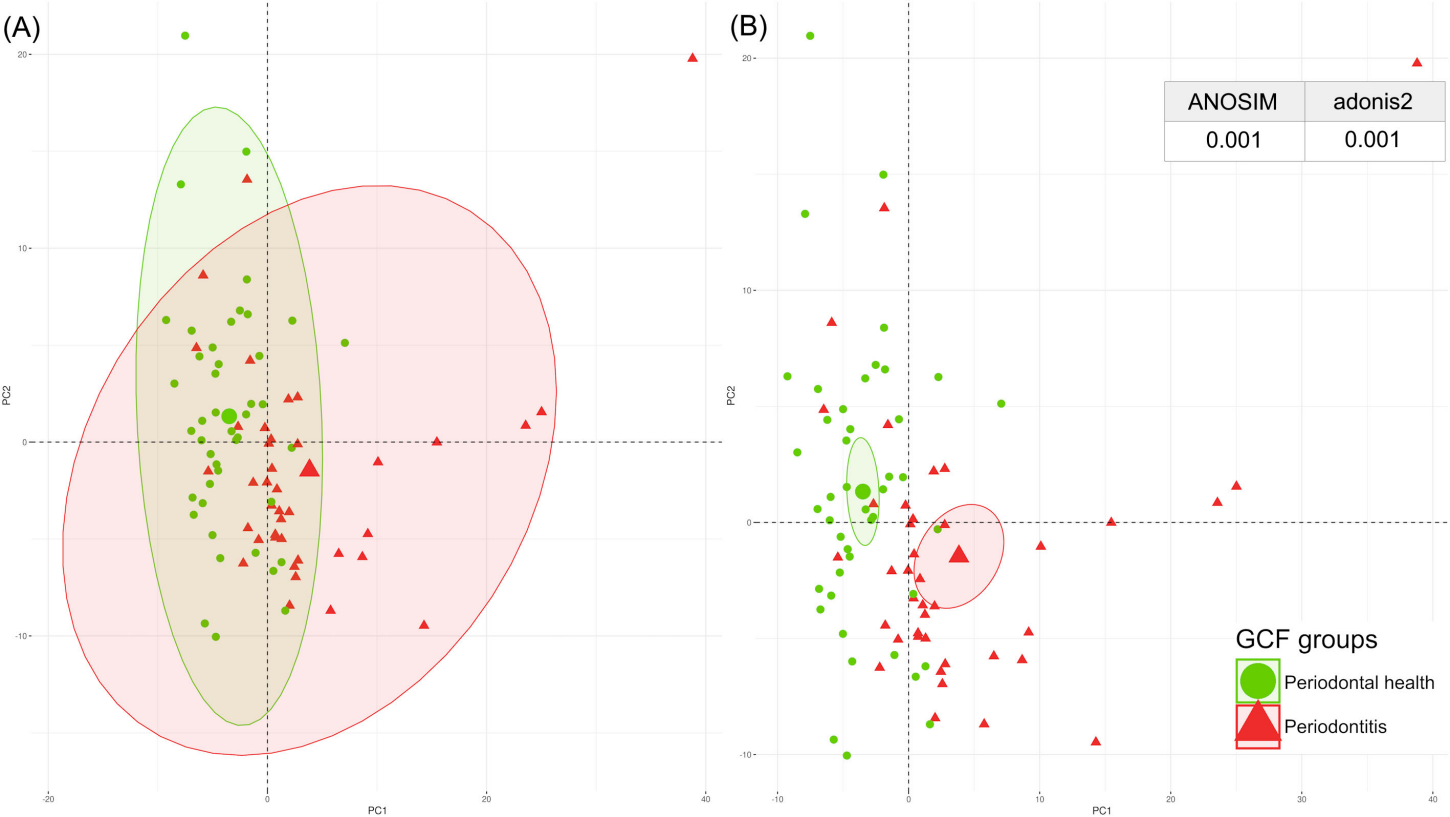
Spatial distribution of the GCF periodontal proteome by PCA, with p-values from ANOSIM and adonis2 tests categorised by periodontal health (green) and periodontitis (red). **(A)** Centroids of each clinical condition with their corresponding ranges of values; **(B)** Centroids of each clinical condition with their corresponding 95% confidence intervals. ANOSIM, analysis of similarities; GCF, gingival crevicular fluid; PCA, principal component analysis. A significant p-value of <0.05 was established.

**Figure 3:**
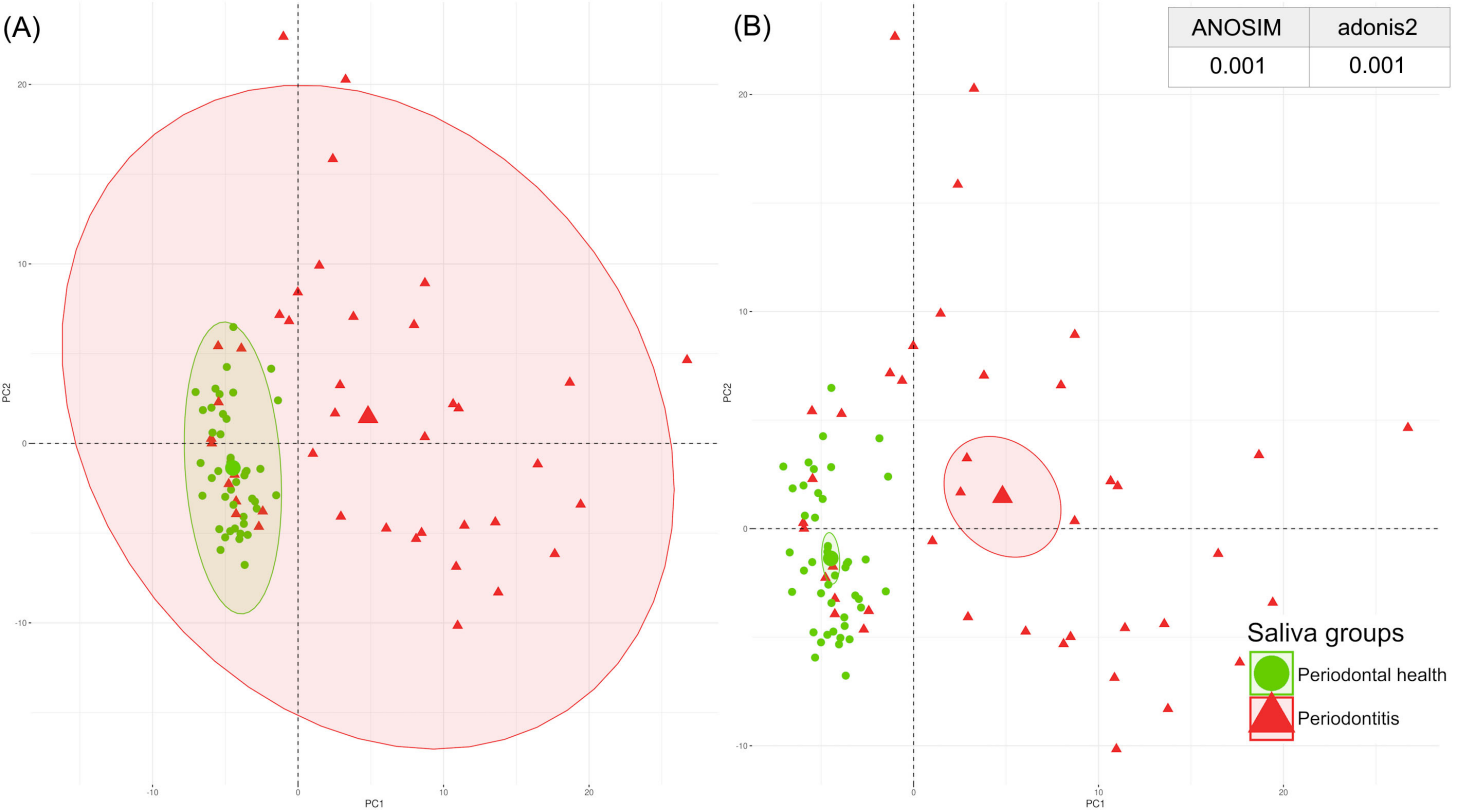
Spatial distribution of the saliva periodontal proteome by PCA with p-values from ANOSIM and adonis2 tests categorised by periodontal health (green) and periodontitis (red). **(A)** Centroids of each clinical condition with their corresponding ranges of values; **(B)** Centroids of each clinical condition with their corresponding 95% confidence intervals. ANOSIM, analysis of similarities; PCA, principal component analysis. A significant p-value of <0.05 was established.

The PCA plot of the GCF and saliva samples revealed distinct clustering between samples with good periodontal health and those with periodontitis. These findings were confirmed by ANOSIM and adonis2 tests, which showed significant differences in GCF and saliva between individuals with periodontitis and those with healthy periodontium (**Figures 2**, **3**).

### Differential expression of the GCF proteins

**Figure 4:**
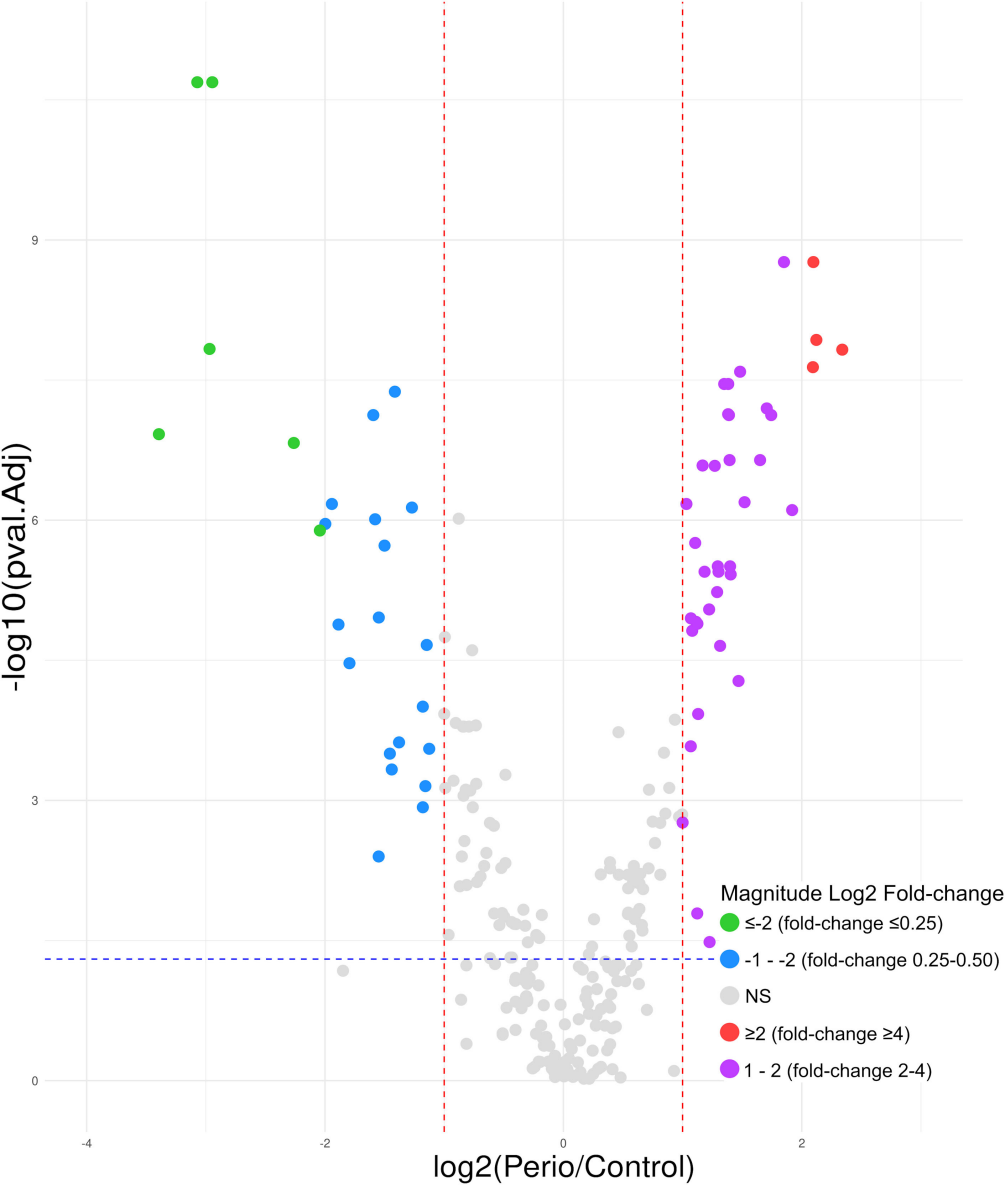
Volcano plot of the 63 GCF proteins differentially expressed in periodontitis compared to periodontal health; 38 upregulated (log2 fold-change ≥2 [red]= 4; 1 − 2 [purple]= 34) and 25 downregulated (log2 fold-change ≤-2 [green]= 6; -1 − -2 [blue]= 19). GCF, gingival crevicular fluid; log, logarithmic; NS, not significant. Differentially expressed proteins were considered if the adjusted p-value was <0.05 (−log10 p-value=1.30) (blue horizontal line) and a fold-change ≥2 (log2 fold-change ≥1) or ≤0.5 (log2 fold-change ≤-1) (red vertical lines). Magnitude Log2 fold-change: ≥2 (red)=fold-change ≥4; 1 − 2 (purple)=fold-change 2 − 4; ≤-2 (green)=fold-change ≤0.25; -1 − -2 (blue)=fold-change 0.25 − 0.50.

Considering the expression of the 250 GCF proteins quantified and the previously established criteria, 63 (25.2%) were found to be differentially expressed in periodontitis compared to periodontal health. Of these, 38 (60.3%) were shown to be upregulated, and 25 (39.7%) were downregulated (**Figure 4**).

Among the 38 overexpressed proteins, four showed a ≥4-fold higher expression in periodontitis compared to periodontal health (log2 fold-change ≥2). Of these, haemoglobin subunits (Hb) beta and alpha showed the highest fold-change (5.06 and 4.35, respectively), followed by carbonic anhydrase 1 (4.28) and protein S100-P (4.27). The remaining 34 proteins showed at least 2-fold higher expression in periodontitis, highlighting immunoglobulins (Ig) heavy constant gamma 3 (3.78), kappa light chain (2.19), and heavy variable 5-51 (2.18), plasma protease C1 inhibitor (3.61), haptoglobin (3.35), and another Hb, delta (3.26) (**Table 2**).

**Table 2:** GCF proteins differentially upregulated in periodontitis compared to periodontal health (N=38 proteins).

UniProt accession	Protein name	Adjusted p-value	Effect size	Fold-change	Mag Log2 fold-change
P68871	Hb beta	1.4938E-08	0.62	5.06	≥2 (N=4)
P69905	Hb alpha	1.1748E-08	0.60	4.35	
P00915	Carbonic anhydrase 1	1.7237E-09	0.71	4.28	
P25815	Protein S100-P	2.3003E-08	1.59	4.27	
P01860	Ig heavy constant gamma 3	7.795E-07	1.17	3.78	1 to− 2 (N=34)
P05155	Plasma protease C1 inhibitor	1.7237E-09	1.32	3.61	
P00738	Haptoglobin	7.4947E-08	1.08	3.35	
P02042	Hb delta	6.3584E-08	0.64	3.26	
P02768	Albumin	2.2729E-07	0.90	3.14	
P43652	Afamin	6.4177E-07	1.04	2.87	
P01008	Antithrombin-III	2.5737E-08	1.27	2.79	
P02730	Band 3 anion transport protein	5.2674E-05	0.73	2.77	
P02749	Beta-2-glycoprotein 1	3.809E-06	0.89	2.65	
P00918	Carbonic anhydrase 2	3.1194E-06	0.46	2.64	
P02774	Vitamin D-binding protein	2.2729E-07	0.94	2.63	
P32119	Peroxiredoxin-2	7.4947E-08	0.61	2.62	
P08697	Alpha-2-antiplasmin	3.4816E-08	1.36	2.61	
P02766	Transthyretin	7.3368E-08	1.17	2.61	
P02751	Fibronectin	3.4816E-08	1.33	2.55	
P19823	ITI H2	2.2156E-05	0.82	2.49	
Q14624	ITI H4	3.5623E-06	0.99	2.46	
P02765	Alpha-2-HS-glycoprotein	3.1194E-06	1.09	2.45	
P01042	Kininogen-1	5.8831E-06	0.96	2.44	
P01023	Alpha-2-macroglobulin	2.6176E-07	1.23	2.41	
P10909	Clusterin	0.0329	0.66	2.34	
P00751	Complement factor B	9.0221E-06	0.93	2.33	
P02679	Fibrinogen gamma chain	3.5623E-06	1.06	2.27	
P01011	Alpha-1-antichymotrypsin	2.5979E-07	1.35	2.25	
P0DOX7	Ig kappa light chain	0.0001	0.89	2.19	
A0A0C4DH38	Ig heavy variable 5-51	0.0162	0.76	2.18	
P13716	Delta-aminolevulinic acid dehydratase	1.2837E-05	0.77	2.18	
P02787	Serotransferrin	1.2263E-05	0.93	2.16	
P0C0L5	Complement C4-B	1.7525E-06	1.06	2.15	
P01024	Complement C3	1.527E-05	0.98	2.12	
P04004	Vitronectin	1.1241E-05	1.08	2.10	
P02790	Hemopexin	0.0003	0.84	2.10	
P01009	Alpha-1-antitrypsin	6.7049E-07	1.10	2.05	
P19827	ITI H1	0.0017	0.68	2.00	

Log, logarithmic; Mag, magnitude; N, number.

Abbreviations related to protein name: Hb, haemoglobin subunit; Ig, immunoglobulin; ITI, inter-alpha-trypsin inhibitor heavy chain.

Differentially upregulated proteins were considered if the adjusted p-value was <0.05 and a fold-change ≥2 (log2 fold-change ≥1).

Magnitude Log2 fold-change: ≥2 (red)=fold-change ≥4; 1 to 2 (purple)=fold-change 2 − 4.

Interestingly, of the 25 downregulated proteins, 14 were keratins, accounting for 56.0%. Considering a fold-change ≤0.25 (log2 fold-change ≤-2), which means a ≤4-fold lower expression in periodontitis, four were keratins: type II cytoskeletal 6B (0.10), type II cytoskeletal 6A (0.13), type II cytoskeletal 5 (0.21), and type I cytoskeletal 16 (0.24). The remaining 11 downregulated keratins exhibited a fold-change of 0.25-0.44. Other protein types that showed a fold-change ≤0.25 were glyceraldehyde-3-phosphate dehydrogenase (GAPDH) (0.12) and zymogen granule protein 16 homolog B (ZG16B) (0.13) (**Table 3**).

**Table 3:** GCF proteins differentially downregulated in periodontitis compared to periodontal health (N=25 proteins).

UniProt accession	Protein name	Adjusted p-value	Effect size	Fold-change	Mag Log2 fold-change
P04259	Keratin, type II cytoskeletal 6B	1.2006E-07	-0.84	0.10	≤-2 (N=6)
P04406	GAPDH	2.0474E-11	-1.72	0.12	
P02538	Keratin, type II cytoskeletal 6A	1.4675E-08	-0.85	0.13	
Q96DA0	ZG16B	2.0474E-11	-1.59	0.13	
P13647	Keratin, type II cytoskeletal 5	1.4901E-07	-0.82	0.21	
P08779	Keratin, type I cytoskeletal 16	1.2894E-06	-0.84	0.24	
P02533	Keratin, type I cytoskeletal 14	1.0958E-06	-0.89	0.25	-1 − to -2 (N=19)
Q6P5S2	Protein LEG1 homolog	6.7049E-07	-0.87	0.26	
P13646	Keratin, type I cytoskeletal 13	1.3100E-05	-0.72	0.27	
P19013	Keratin, type II cytoskeletal 4	3.3994E-05	-0.72	0.29	
P04264	Keratin, type II cytoskeletal 1	7.4947E-08	-0.77	0.33	
P01833	Polymeric Ig receptor	9.7776E-07	-0.81	0.33	
P48668	Keratin, type II cytoskeletal 6C	0.0040	-0.71	0.34	
P07355	Annexin A2	1.1010E-05	-0.80	0.34	
P13645	Keratin, type I cytoskeletal 10	1.8719E-06	-0.61	0.35	
Q04695	Keratin, type I cytoskeletal 17	0.0003	-0.76	0.36	
P08185	Corticosteroid-binding globulin	0.0005	-0.76	0.37	
P49913	Cathelicidin antimicrobial peptide	4.2083E-08	-1.34	0.38	
Q01546	Keratin, type II cytoskeletal 2 oral	0.0002	-0.51	0.38	
P35527	Keratin, type I cytoskeletal 9	7.3206E-07	-0.65	0.41	
P35908	Keratin, type II cytoskeletal 2 epidermal	9.9088E-05	-0.49	0.44	
P20700	Lamin-B1	0.0012	-0.71	0.44	
P02545	Prelamin-A/C	0.0007	-0.93	0.45	
P08865	40S ribosomal protein SA	2.1657E-05	-0.96	0.45	
P19961	Alpha-amylase 2B	0.0003	-0.72	0.46	

Log, logarithmic; Mag, magnitude; N, number.

Abbreviations related to protein name: GAPDH, glyceraldehyde-3-phosphate dehydrogenase; Ig, immunoglobulin; LEG, liver-enriched gene; ZG16B, zymogen granule protein 16 homolog B.

Differentially downregulated proteins were considered if the adjusted p-value was <0.05 and a fold-change ≤0.50 (log2 fold-change ≤-1).

Magnitude Log2 fold-change: ≤-2 (green)=fold-change ≤0.25; -1 to -2 (blue)=fold-change 0.25 − 0.50.

### Differential expression of the salivary proteins

**Figure 5:**
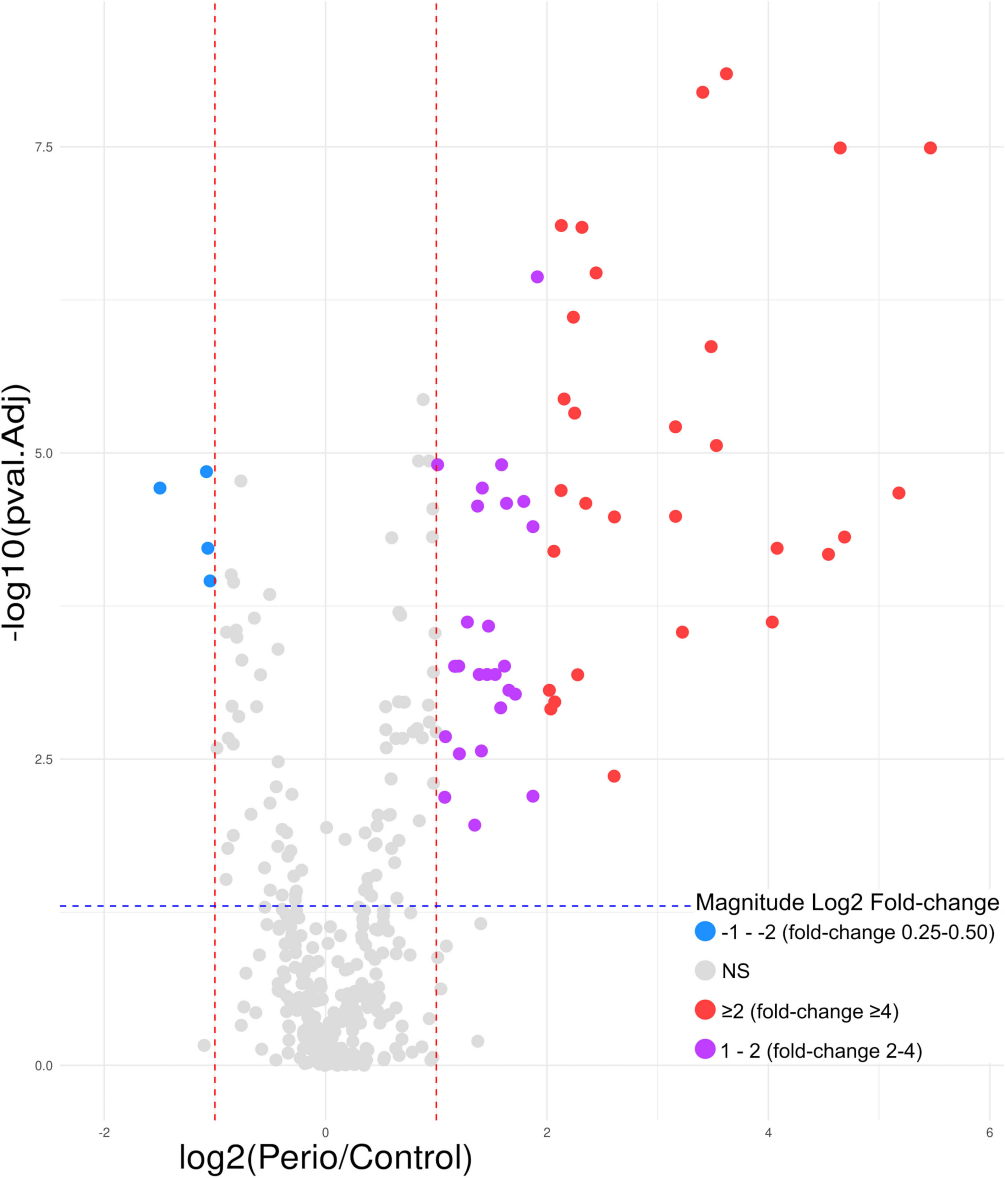
Volcano plot of the 59 saliva proteins differentially expressed in periodontitis compared to periodontal health; 55 upregulated (log2 fold-change ≥2 [red]= 29; 1 − 2 [purple]= 26) and 4 downregulated (log2 fold-change -1 − -2 [blue]). log, logarithmic; NS, not significant. Differentially expressed proteins were considered if the adjusted p-value was <0.05 (−log10 p-value=1.30) (blue horizontal line) and a fold-change ≥2 (log2 fold-change ≥1) or ≤0.5 (log2 fold-change ≤-1) (red vertical lines). Magnitude Log2 fold-change: ≥2 (red)=fold-change ≥4; 1 − 2 (purple)=fold-change 2 − 4; -1 − -2 (blue)=fold-change 0.25 − 0.50.

In saliva, of the 377 proteins quantified, 59 (15.7%) were differentially expressed, with 55 (93.2%) being upregulated and only four (6.8%) downregulated (**Figure 5**).

Consequently, the most differentially expressed proteins were upregulated, with beta-2-microglobulin exhibiting the greatest fold-change (44.14). Furthermore, in contrast to GCF, nine keratins were overexpressed in saliva in periodontitis, with seven exhibiting a fold-change of ≥ 4 and two between 2 and 4. Of these, keratin, type I cytoskeletal 13 showed the highest fold-change (36.23), and the remaining eight values ranged from 2.65 to 23.31. Other noteworthy proteins include the S100 family: A9 (12.30), A8 (10.61), A12 (4.76), P (4.72), and A6 (2.67), in addition to Ig heavy constant alpha 1 (4.45), alpha-2 heavy chain (2.59), and Hb beta (4.20) and alpha (4.06). Considering the remaining overexpressed proteins, of particular interest were neutrophil defensin 1 (25.08), annexin A1 (9.34), lysozyme C (4.98), resistin (4.37), cathepsin G (3.28), myeloperoxidase (2.77), and azurocidin (2.75) (**Table 4**).

**Table 4:** Salivary proteins differentially upregulated in periodontitis compared to periodontal health (N=55 proteins).

UniProt accession	Protein name	Adjusted p-value	Effect size	Fold-change	Mag Log2 fold-change
P61769	Beta-2-microglobulin	3.2279E-08	1.45	44.14	≥2 (N=29)
P13646	Keratin, type I cytoskeletal 13	2.1223E-05	1.20	36.23	
Q9UBC9	Small proline-rich protein 3	4.8555E-05	0.58	25.77	
P59665	Neutrophil defensin 1	3.2279E-08	0.76	25.08	
P13647	Keratin, type II cytoskeletal 5	6.7217E-05	1.16	23.31	
Q01546	Keratin, type II cytoskeletal 2 oral	5.9948E-05	1.17	16.90	
P19013	Keratin, type II cytoskeletal 4	0.0002	1.20	16.39	
P06702	Protein S100-A9	8.006E-09	0.97	12.30	
P08779	Keratin, type I cytoskeletal 16	8.6934E-06	1.18	11.55	
P04264	Keratin, type II cytoskeletal 1	1.3533E-06	1.62	11.18	
P05109	Protein S100-A8	1.1339E-08	1.08	10.61	
P04083	Annexin A1	0.0003	1.20	9.34	
P10599	Thioredoxin	3.2903E-05	1.44	8.95	
P28676	Grancalcin	6.1142E-06	0.84	8.94	
Q04695	Keratin, type I cytoskeletal 17	3.327E-05	1.02	6.10	
O14818	Proteasome subunit alpha type-7	0.0044	1.04	6.09	
Q02487.2	Isoform 2 of desmocollin-2	3.3862E-07	1.17	5.44	
P07305	Histone H1.0	2.5758E-05	0.81	5.10	
P61626	Lysozyme C	1.4364E-07	1.04	4.98	
P04792	Heat shock protein beta-1	0.0006	1.02	4.85	
P80511	Protein S100-A12	4.7261E-06	1.01	4.76	
P25815	Protein S100-P	7.7803E-07	1.29	4.72	
P01876	Ig heavy constant alpha 1	3.6244E-06	1.06	4.45	
Q9HD89	Resistin	1.3885E-07	0.91	4.37	
P19105	Myosin regulatory light chain 12A	2.0242E-05	1.25	4.37	
P68871	Hb beta	0.0011	0.58	4.20	
Q9UBG3	Cornulin	6.3468E-05	0.99	4.18	
P02652	Apolipoprotein A-II	0.0012	0.75	4.10	
P69905	Hb alpha	0.0009	0.55	4.06	
Q9NUQ9	CYFIP-related Rac1 interactor B	3.6474E-07	1.25	3.77	1 to− 2 (N=26)
P04114	Apolipoprotein B-100	3.9948E-05	0.87	3.66	
P08865	40S ribosomal protein SA	0.0063	0.93	3.66	
P62805	Histone H4	2.4866E-05	1.18	3.46	
P08311	Cathepsin G	0.0009	0.77	3.28	
P0DUB6	Alpha-amylase 1A	0.0009	0.89	3.15	
Q8N1N4	Keratin, type II cytoskeletal 78	2.5758E-05	1.21	3.10	
P60842	Eukaryotic initiation factor 4A-I	0.0005	1.20	3.07	
P02671	Fibrinogen alpha chain	1.2471E-05	1.00	3.01	
P07355	Annexin A2	0.0012	1.14	2.99	
P02647	Apolipoprotein A-I	0.0006	0.71	2.89	
P05164	Myeloperoxidase	0.0003	0.89	2.77	
P20160	Azurocidin	0.0006	0.75	2.75	
P06703	Protein S100-A6	1.9327E-05	1.19	2.67	
P08727	Keratin, type I cytoskeletal 19	0.0027	0.84	2.65	
P12429	Annexin A3	0.0006	0.85	2.62	
P0DOX2	Ig alpha-2 heavy chain	2.7128E-05	0.99	2.59	
P68431	Histone H3.1	0.0109	0.32	2.54	
P62987	Ubiquitin-60S ribosomal protein L40	0.0002	1.28	2.43	
Q9BTM1	Histone H2A.J	0.0029	0.83	2.31	
P49913	Cathelicidin antimicrobial peptide	0.0005	1.01	2.30	
Q99879	Histone H2B type 1-M	0.0006	0.93	2.26	
P43490	Nicotinamide phosphoribosyltransferase	0.0006	1.03	2.24	
P13639	Elongation factor 2	0.0021	1.14	2.12	
P62258	14-3–3 protein epsilon	0.0065	0.91	2.11	
P35579	Myosin-9	1.2471E-05	1.23	2.02	

Log, logarithmic; Mag, magnitude; N, number. Abbreviations related to protein name: CYFIP, cytoplasmic fragile X messenger ribonucleoprotein 1-interacting protein; Hb, haemoglobin subunit; Ig, immunoglobulin.

Differentially upregulated proteins were considered if the adjusted p-value was <0.05 and a fold-change ≥2 (log2 fold-change ≥1).

Magnitude Log2 fold-change: ≥2 (red)=fold-change ≥4; 1 to 2 (purple)=fold-change 2 − 4.

The fold-change of the downregulated proteins was considerably lower, with no protein having a value of ≤0.25. The most underexpressed protein was lipocalin-1 with a fold-change of 0.35 (**Table 5**).

**Table 5:** Salivary proteins differentially downregulated in periodontitis compared to periodontal health (N=4 proteins).

UniProt accession	Protein name	Adjusted p-value	Effect size	Fold-change	Mag Log2 fold-change
P31025	Lipocalin-1	1.9327E-05	-0.81	0.35	-1 − to -2 (N=4)
P30086	Phosphatidylethanolamine-binding protein 1	1.4203E-05	-1.05	0.47	
P10809	60 kDa heat shock protein, mitochondrial	5.9948E-05	-0.62	0.48	
Q96DA0	ZG16B	0.0001	-0.76	0.49	

Log, logarithmic; Mag, magnitude; N, number.

Abbreviations related to protein name: kDa, kilodalton; ZG16B, zymogen granule protein 16 homolog B.

Differentially downregulated proteins were considered if the adjusted p-value was <0.05 and a fold-change ≤0.50 (log2 fold-change ≤-1).

Magnitude Log2 fold-change: -1 to -2 (blue)=fold-change 0.25 − 0.50.

### Comparison of differentially expressed proteins in GCF and saliva

Comparing the results obtained in GCF and saliva, 14 proteins were differentially expressed in both sample types, representing 22.2% and 23.7% of the total proteins differentially expressed, respectively.

As previously noted, Hb beta and alpha were overexpressed in GCF and saliva (GCF: 5.06 and 4.35; saliva: 4.20 and 4.06, respectively), in addition to protein S100-P (4.27; 4.72). Furthermore, ZG16B was found to be downregulated in both sample types (0.13; 0.49).

However, the remaining 10 proteins were underexpressed in GCF but overexpressed in saliva. Interestingly, seven of these proteins were keratins (types I cytoskeletal 13, 16, and 17, and types II cytoskeletal 1, 2 oral, 4, and 5) with a fold-change in GCF of 0.21-0.38, whereas in saliva it was 6.10-36.23. The remaining three proteins were annexin A2 (0.34; 2.99), cathelicidin antimicrobial peptide (0.38; 2.30), and 40S ribosomal protein SA (0.45; 3.66) (**Table 6**).

**Table 6:** Proteins differentially expressed in GCF and saliva in periodontitis compared to periodontal health (N=14 proteins).

UniProt accession	Protein name	GCF			Saliva		
		Adjusted p-value	Fold-change	Mag.	Adjusted p-value	Fold-change	Mag.
P68871	Hb beta	1.4938E-08	5.06	UP	0.0011	4.20	UP
P69905	Hb alpha	1.1748E-08	4.35		0.0009	4.06	
P25815	Protein S100-P	2.3003E-08	4.27		7.7803E-07	4.72	
Q96DA0	ZG16B	2.0474E-11	0.13	DW	0.0001	0.49	DW
P13647	Keratin, type II cytoskeletal 5	1.4901E-07	0.21		6.7217E-05	23.31	UP
P08779	Keratin, type I cytoskeletal 16	1.2894E-06	0.24		8.6934E-06	11.55	
P13646	Keratin, type I cytoskeletal 13	1.3100E-05	0.27		2.1223E-05	36.23	
P19013	Keratin, type II cytoskeletal 4	3.3994E-05	0.29		0.0002	16.39	
P04264	Keratin, type II cytoskeletal 1	7.4947E-08	0.33		1.3533E-06	11.18	
Q04695	Keratin, type I cytoskeletal 17	0.0003	0.36		3.327E-05	6.10	
Q01546	Keratin, type II cytoskeletal 2 oral	0.0002	0.38		5.9948E-05	16.90	
P07355	Annexin A2	1.1010E-05	0.34		0.0012	2.99	
P49913	Cathelicidin antimicrobial peptide	4.2083E-08	0.38		0.0005	2.30	
P08865	40S ribosomal protein SA	2.1657E-05	0.45		0.0063	3.66	

DW, downregulated; GCF, gingival crevicular fluid; Mag, magnitude of fold-change; UP, upregulated.

Abbreviations related to protein name: Hb, haemoglobin subunit; ZG16B, zymogen granule protein 16 homolog B.

Differentially proteins were considered if the adjusted p-value was <0.05 and a fold-change ≥2 (log2 fold-change ≥1; upregulated) or ≤0.5 (log2 fold-change ≤-1; downregulated).

## Discussion

The analysis of GCF and saliva samples using SWATH-MS enabled the quantification of 250 abundant proteins in GCF and 377 in saliva. In both fluids, the structure of the periodontal proteome differed between individuals with periodontitis and those with a healthy periodontium. Regarding protein expression, approximately 25% of GCF proteins and 16% of salivary proteins were found to be differentially expressed. In both fluids, most of these proteins were overexpressed in periodontitis—60% vs. 40% in GCF and 93% vs. 7% in saliva, respectively. Furthermore, although 95% of GCF proteins and 63% of salivary proteins were quantified in both sample types, the most differentially expressed proteins varied, and only about 22% of them were shared between the two oral fluids.

### Methodological differences in periodontal proteome analysis

To date, the periodontal proteome has been evaluated using different proteomic techniques (Rizal et al., 2020). However, this is the first study to use SWATH-MS to analyse the structure and differential protein expression of the periodontal proteome.

Sample size is a relevant limitation in previous proteomics research, with most studies including ≤20 subjects per clinical group (Bellei et al., 2022; Bostanci et al., 2018; Casarin et al., 2023; Grant et al., 2022; Guzman et al., 2018; Mertens et al., 2018; Tang et al., 2019; Yi et al., 2021). In contrast, the present study included a notably larger sample size, with more than 40 patients. Increasing the sample size is crucial for enhancing statistical robustness and improving the ability to detect true differences in protein expression levels between clinical conditions (Ahmad et al., 2025).

The periodontal proteome has been primarily studied in gingival crevicular fluid (GCF) and saliva samples (Guzman et al., 2014), as both oral fluids are valuable for investigating the oral and systemic environments (Ahmad et al., 2025). However, there is a tendency to justify the selection of one fluid over the other, since most studies have evaluated the periodontal proteome in either GCF or saliva (Bellei et al., 2022; Bostanci et al., 2018; Casarin et al., 2023; Hartenbach et al., 2020; Kim et al., 2021; Yi et al., 2021). Only two studies have analysed both sample types (Grant et al., 2022; Tang et al., 2019). These studies focused on assessing the diagnostic accuracy of proteins without comparing the structural and expression differences between the GCF and saliva proteomes.

Analysis of GCF and saliva samples using SWATH-MS yielded a spectral library of 427 proteins, with 250 and 377 proteins quantified in each fluid, respectively, based on stringent criteria that required at least 10 peptides per protein and seven fragments per peptide. Although this number of quantified proteins is lower compared to previous studies, those studies applied less stringent criteria, quantifying proteins based on as few as two unique peptides (Bostanci et al., 2018; Grant et al., 2022; Kim et al., 2021; Silbereisen et al., 2024; Yi et al., 2021).

Although we could have employed more extensive libraries or adopted a library-free DIA approach (Demichev et al., 2020; Rosenberger et al., 2014), building our own spectral library ensured accurate protein identification by focusing on the most abundant proteins. This approach lays the groundwork for further validation using less sensitive methods, which could ultimately support clinical applications.

Comparing GCF and saliva, saliva samples have a higher number of proteins (Grant et al., 2022; Tang et al., 2019). This is because saliva also comprises proteins from the salivary glands (Guzman et al., 2014). Moreover, 238 proteins were common to both oral fluids, representing 95% of those in GCF and 63% in saliva. This reflects that most GCF components are excreted from the pocket through saliva and can be identified in both fluids (Bostanci and Bao, 2017).

Grant et al. reported that about 30% of the total proteins were present in both fluids (Grant et al., 2022). This lower proportion may be attributed to the different proteomic techniques used (isobaric tag for relative and absolute quantitation -iTRAQ-) and the analysis of stimulated saliva samples (Grant et al., 2022). Conversely, we collected unstimulated saliva as the stimulation of salivary flow may produce inappropriate glycoprotein glycosylation (Buzalaf et al., 2020).

The criteria applied in previous publications to consider a protein as differentially expressed were based on a p-value <0.05, a minimum fold-change, or both (Bellei et al., 2022; Bostanci et al., 2018; Casarin et al., 2023; Hartenbach et al., 2020; Kim et al., 2021; Shin et al., 2019). However, our study was more demanding, being the first to apply the Benjamini-Hochberg correction for controlling false positives (Benjamini and Hochberg, 1995). This variability in the criteria applied, as well as the different proteomics techniques employed, complicates the comparison of the results obtained with other studies.

Using a more permissive statistical threshold may increase the number of differentially expressed proteins detected. However, in scenarios involving distinct clinical states—such as periodontal health and advanced periodontitis—it is more relevant to prioritise the most strongly differentially expressed proteins. Conversely, when comparing closely related conditions, such as periodontal health and gingivitis, applying a less stringent threshold may be necessary to detect subtle variations in protein expression.

### Periodontal proteome structure in GCF and saliva

The periodontal proteome structure was different between periodontitis and periodontal health in both sample types, consistent with previous research (Shin et al., 2019; Yi et al., 2021). Our research group demonstrated that smoking status does not alter the distribution of the GCF periodontal proteome in periodontitis (Blanco-Pintos et al., 2025b). Therefore, periodontitis is a more relevant condition for modifying the structure of the periodontal proteome. However, future research should investigate the impact of other clinical variables, such as age, on the spatial distribution of the periodontal proteome.

### Differential expression of GCF proteins

The functions and possible relationship to periodontitis of the most relevant GCF proteins differentially expressed are specified in **Table 7**.

**Table 7:** Functions and relationship to periodontitis of the most relevant GCF proteins differentially expressed in periodontitis compared to periodontal health.

Mag	UniProt accession	Protein name	Relation with periodontitis
UPREGULATED	P68871	Hb beta	Bleeding caused by inflammation (Guzman et al., 2014)
	P69905	Hb alpha	
	P02042	Hb delta	
	P00915	Carbonic anhydrase 1	Unclear
	P25815	Protein S100-P	Unclear
	P01860	Ig heavy constant gamma 3	Bacterial defence line (Megha and Mohanan, 2021)
	P0DOX7	Ig kappa light chain	
	A0A0C4DH38	Ig heavy variable 5-51	
	P05155	Plasma protease C1 inhibitor	Antiinflammatory activity (Beinrohr et al., 2011)
	P00738	Haptoglobin	Bind free haemoglobin. Acute phase protein (Smith and McCulloh, 2015)
DOWNREGULATED	P04259	Keratin, type II cytoskeletal 6B	Maintain epithelial integrity (Groeger and Meyle, 2019)
	P02538	Keratin, type II cytoskeletal 6A	
	P13647	Keratin, type II cytoskeletal 5	
	P08779	Keratin, type I cytoskeletal 16	
	P02533	Keratin, type I cytoskeletal 14	
	P13646	Keratin, type I cytoskeletal 13	
	P19013	Keratin, type II cytoskeletal 4	
	P04264	Keratin, type II cytoskeletal 1	
	P48668	Keratin, type II cytoskeletal 6C	
	P13645	Keratin, type I cytoskeletal 10	
	Q04695	Keratin, type I cytoskeletal 17	
	Q01546	Keratin, type II cytoskeletal 2 oral	
	P35527	Keratin, type I cytoskeletal 9	
	P35908	Keratin, type II cytoskeletal 2 epidermal	
	P04406	GAPDH	GAIT complex (Sampath et al., 2003)
	Q96DA0	ZG16B	Unclear

GAIT, gamma-interferon-activated inhibitor of translation; Mag, magnitude of fold-change.

Abbreviations related to protein name: GAPDH, glyceraldehyde-3-phosphate dehydrogenase; Hb, haemoglobin subunit; Ig, immunoglobulin; ZG16B, zymogen granule protein 16 homolog B.

Differentially upregulated proteins were considered if the adjusted p-value was <0.05 and a fold-change ≥2 (log2 fold-change ≥1) or ≤0.5 (log2 fold-change ≤-1).

Hb beta and alpha were the most overexpressed proteins, with Hb delta also being up to five times increased in periodontitis. It is reported that, despite discarding highly visible blood-contaminated GCF samples, there is a greater tendency for spontaneous bleeding in periodontitis due to inflammation of periodontal tissues (Guzman et al., 2014).

Consequently, haptoglobin was also found to be upregulated (fold-change >3), because it binds to free haemoglobin. Furthermore, haptoglobin is an acute-phase protein, and its expression is increased in infectious and inflammatory processes (Smith and McCulloh, 2015). Previous proteomic studies have also demonstrated the overexpression of these proteins in periodontitis (Bellei et al., 2022; Carneiro et al., 2014; Tang et al., 2019).

Moreover, the Ig’s heavy constant gamma 3, kappa light chain, and heavy variable 5–51 were also shown to be upregulated (fold-change >2). Igs are essential components of the immune system for recognising and neutralising pathogens by binding to their antigens (Megha and Mohanan, 2021). Therefore, in periodontitis, their expression is increased due to bacterial dysbiosis and inflammation of periodontal tissues (Bellei et al., 2022; Carneiro et al., 2014; Marinho et al., 2019; Yi et al., 2021).

Considering the remaining upregulated proteins, the plasma protease C1 inhibitor (fold-change >3.5) is particularly noteworthy. This protein functions as an essential antiinflammatory protein by regulating the complement system (Beinrohr et al., 2011). However, carbonic anhydrase 1 and protein S100-P, they were overexpressed in periodontitis (fold-change around 4.3 for both), as in previous studies (Baliban et al., 2012; Silva-Boghossian et al., 2013); however, their relationship with periodontitis is still unclear.

Conversely, the most differentially expressed proteins were downregulated, as keratin, type II cytoskeletal 6B showed a fold-change of 0.10, implying a 10-fold lower expression in periodontitis compared to periodontal health. Moreover, the majority (56%) of the 25 proteins underexpressed in periodontitis were different types of keratins. Keratins are essential structural proteins in the epithelial barrier, providing resistance to microbial invasion (Groeger and Meyle, 2019). The decrease in keratins in GCF periodontitis samples may be related to periodontal tissue degradation, compromising the integrity of the epithelium and making the tissues more vulnerable to bacterial invasion (Groeger and Meyle, 2019).

Apart from keratins, the most underexpressed proteins include GAPDH and ZG16B with also a fold-change of around 0.10. GAPDH is a key enzyme in glycolysis (Tisdale, 2002), but it is also part of the gamma interferon-activated inhibitor of translation (GAIT) complex. This complex may contribute to the resolution of the inflammatory response following cytokine activation (Sampath et al., 2003). Therefore, its underexpression may be associated with reduced antiinflammatory activity of the GAIT complex. However, the function of ZG16B and its potential relationship with periodontitis remain unclear.

### Differential expression of the salivary proteins

In saliva, the most differentially expressed protein was beta-2-microglobulin, which was approximately 44 times more abundant in patients with periodontitis than in healthy subjects. This protein is a component of major histocompatibility complex (MHC) class I molecules and is released during inflammatory processes due to its crucial role in the immune system and antigen presentation (Bernier, 1980; Sreejit et al., 2014) (**Table 8**).

**Table 8:** Functions and relationship to periodontitis of the most relevant salivary proteins differentially expressed in periodontitis compared to periodontal health.

Mag	UniProt accession	Protein name	Relation with periodontitis
UPREGULATED	P61769	Beta-2-microglobulin	Presentation of antigens and regulator of the immune system (Bernier, 1980; Sreejit et al., 2014)
	P59665	Neutrophil defensin 1	Antibacterial activity, induces the production of proinflammatory cytokines (Ericksen et al., 2005; Wang et al., 2016)
	P04083	Annexin A1	Control proinflammatory stimuli (Casarin et al., 2023)
	P61626	Lysozyme C	Antibacterial activity, enhances the activity of immunoagents (Surna et al., 2009)
	Q9HD89	Resistin	Stimulate proinflammatory reaction (Tripathi et al., 2020)
	P08311	Cathepsin G	Antibacterial activity, regulate inflammatory reaction (Gao et al., 2018)
	P05164	Myeloperoxidase	Antimicrobial and anti/proinflammatory activity (Aratani, 2018)
	P20160	Azurocidin	Antimicrobial activity, induces cytokine release (Soehnlein and Lindbom, 2009)
	P13646	Keratin, type I cytoskeletal 13	Maintain epithelial integrity (Groeger and Meyle, 2019)
	P13647	Keratin, type II cytoskeletal 5	
	Q01546	Keratin, type II cytoskeletal 2 oral	
	P19013	Keratin, type II cytoskeletal 4	
	P08779	Keratin, type I cytoskeletal 16	
	P04264	Keratin, type II cytoskeletal 1	
	Q04695	Keratin, type I cytoskeletal 17	
	Q8N1N4	Keratin, type II cytoskeletal 78	
	P08727	Keratin, type I cytoskeletal 19	
	P06702	Protein S100-A9	Heterodimer S100A8/A9 (calprotectin) has antimicrobial and proinflammatory activity (Wang et al., 2018)
	P05109	Protein S100-A8	
	P80511	Protein S100-A12	Innate immune response with proinflammatory function (Meijer et al., 2012)
	P25815	Protein S100-P	Unclear
	P06703	Protein S100-A6	Unclear
	P01876	Ig heavy constant alpha 1	Bacterial defence line (Megha and Mohanan, 2021)
	P0DOX2	Ig alpha-2 heavy chain	
	P68871	Hb beta	Bleeding caused by inflammation (Guzman et al., 2014)
	P69905	Hb alpha	
DW	P31025	Lipocalin-1	Possible relation with innate immunity (Ong et al., 2006).

DW, downregulated; Mag, magnitude of fold-change.

Abbreviations related to protein name: Hb, haemoglobin subunit; Ig, immunoglobulin.

Differentially upregulated proteins were considered if the adjusted p-value was <0.05 and a fold-change ≥2 (log2 fold-change ≥1) or ≤0.5 (log2 fold-change ≤-1).

Other proteins that stand out are neutrophil defensin 1, annexin A1, lysozyme C, resistin, cathepsin G, myeloperoxidase, and azurocidin, which were upregulated between 2.8 and 25.1-fold in periodontitis. These proteins participate in the immune system by having proinflammatory (promoting cytokine secretion) and/or antiinflammatory functions (Aratani, 2018; Casarin et al., 2023; Gao et al., 2018; Soehnlein and Lindbom, 2009; Surna et al., 2009; Tripathi et al., 2020; Wang et al., 2016). Additionally, some of them have also demonstrated antimicrobial activity (Ericksen et al., 2005) (**Table 8**). Thus, overexpression of these proteins can indicate the dysregulation of the host immune response in periodontitis.

Similarly, other proteins with proinflammatory and antimicrobial functions that were overexpressed were those belonging to the S100 family, A8, A9, and A12, with a fold-change >4.5 (Meijer et al., 2012; Wang et al., 2018). Moreover, S100-P and S100-A6 were also found to be upregulated, as in previous studies (Grant et al., 2022; Salazar et al., 2013), although their possible relationship with periodontitis has not yet been discovered.

Furthermore, as in GCF, two Igs (heavy constant alpha 1 and alpha-2 heavy chain), as well as Hb beta and alpha, were overexpressed in periodontitis. However, keratins showed a different expression pattern, as nine keratins were found to be upregulated in saliva.

Conversely, only four salivary proteins were found to be downregulated in periodontitis. Moreover, the difference in expression with respect to healthy subjects was lower than that shown by the overexpressed proteins. Lipocalin-1 was the most underexpressed saliva protein with a nearly three times lower expression in periodontitis. This protein is involved in taste perception but also has a possible relationship with innate immunity (Ong et al., 2006).

### Periodontal proteome expression in GCF and saliva: similarities and differences

As mentioned above, the protein composition of GCF and saliva samples was similar, as 95% of abundant GCF proteins and 63% of abundant salivary proteins were quantified in both oral fluids. However, the proportion of differentially expressed proteins was higher in GCF than in saliva (25% vs. 16%).

Furthermore, although more upregulated than downregulated proteins were found in both sample types in periodontitis, the distribution was more balanced in GCF (60% vs. 40%), whereas saliva showed a strong predominance of overexpressed proteins (93% vs. 7%). Additionally, the most differentially expressed proteins in GCF were underexpressed in periodontitis, with expression levels up to 10-fold lower. In contrast, in saliva, 11 proteins showed a 10-fold or greater increase in expression in periodontitis, with the highest difference reaching a 44-fold increase.

When comparing the differentially expressed proteins in both sample types, keratins are particularly noteworthy, as previously mentioned. Of the 14 proteins differentially expressed in both oral fluids, seven were keratins that were underexpressed in GCF but overexpressed in saliva (**Table 9**). This contrasting pattern may result from keratin exfoliation from the periodontal pocket due to epithelial barrier breakdown, leading to increased concentrations in saliva (Bostanci and Bao, 2017).

**Table 9:** Functions and relationship to periodontitis of the GCF and salivary proteins differentially expressed in periodontitis compared to periodontal health in both oral fluids.

Mag		UniProt accession	Protein name	Relation with periodontitis
GCF	Saliva			
UP	UP	P68871	Hb beta	Bleeding caused by inflammation (Guzman et al., 2014)
		P69905	Hb alpha	
		P25815	Protein S100-P	Unclear
DOWNREGULATED	DW	Q96DA0	ZG16B	Unclear
	UPREGULATED	P13647	Keratin, type II cytoskeletal 5	Maintain epithelial integrity (Groeger and Meyle, 2019)
		P08779	Keratin, type I cytoskeletal 16	
		P13646	Keratin, type I cytoskeletal 13	
		P19013	Keratin, type II cytoskeletal 4	
		P04264	Keratin, type II cytoskeletal 1	
		Q04695	Keratin, type I cytoskeletal 17	
		Q01546	Keratin, type II cytoskeletal 2 oral	
		P07355	Annexin A2	Annexin A2/S100-A10 heterotetramer has proinflammatory function (Bharadwaj et al., 2013)
		P49913	Cathelicidin antimicrobial peptide	Antimicrobial protein, integral component of the innate immune system (Murakami et al., 2004)
		P08865	40S ribosomal protein SA	Facilitate bacteria elimination, antiinflammatory activity (Sun et al., 2021)

DW, downregulated; GCF, gingival crevicular fluid; Mag, magnitude of fold-change; UP, upregulated.

Abbreviations related to protein name: Hb, haemoglobin subunit; ZG16B, zymogen granule protein 16 homolog B.

Differentially upregulated proteins were considered if the adjusted p-value was <0.05 and a fold-change ≥2 (log2 fold-change ≥1) or ≤0.5 (log2 fold-change ≤-1).

A similar opposing expression pattern between GCF and saliva was observed for annexin A2, cathelicidin antimicrobial peptide, and 40S ribosomal protein SA. These proteins play roles in the immune response, exhibiting antiinflammatory, proinflammatory, and antimicrobial activities (Bharadwaj et al., 2013; Murakami et al., 2004; Sun et al., 2021).

### Implications for practice and research perspectives

To better understand the periodontal proteome in GCF and saliva across periodontal health and periodontitis, we included a heterogeneous population with respect to age and smoking status. Proteomic analyses have shown that both factors influence protein expression (Blanco-Pintos et al., 2025b; Zhang et al., 2022). This diversity enhances the generalisability of the findings by helping to isolate the specific impact of periodontitis on the proteome. However, the influence of clinical variables on the periodontal proteome in GCF and saliva should be further investigated. This would allow for a more accurate evaluation and comparison of their effects on the proteome in periodontitis across both oral fluids.

Additionally, we analysed two distinct clinical conditions: periodontal health and advanced periodontitis (stages III–IV). Future research should also investigate the periodontal proteome in earlier disease stages, such as initial periodontitis (stages I–II) and gingivitis, in both GCF and saliva. This would help clarify how the proteome is progressively altered from health to disease, and how these molecular changes are reflected in both oral fluids.

From a clinical perspective, differentially expressed proteins cannot be considered biomarkers without diagnostic accuracy analyses (Gürsoy and Kantarci, 2022), such as those previously published by our research group (Blanco-Pintos et al., 2024, 2025a). Consistently, nearly all proteins demonstrating excellent diagnostic accuracy for detecting periodontitis were differentially expressed in both GCF and saliva. This includes proteins such as ZG16B and carbonic anhydrase 1, whose relationship with periodontitis remains unclear. As a result, functional studies aimed at elucidating the role of these proteins in the pathogenesis of periodontitis are of particular interest.

Moreover, the proteins with the highest diagnostic capacity differed between GCF and saliva, just as the most differentially expressed proteins varied between these fluids. This study is the first to demonstrate that while similar proteins are quantified in both GCF and saliva, their expression patterns differ qualitatively and quantitatively, underscoring the potential need for different molecular biomarkers in each oral fluid.

In previous studies from our research group, the diagnostic accuracy of GCF proteins was found to be higher than that of salivary proteins (Blanco-Pintos et al., 2024, 2025a). This difference may be attributed to the higher protein composition in saliva, which leads to greater inter-subject variability (Guzman et al., 2014). Clinically, both oral fluids are applicable, as while GCF biomarkers are probably more relevant in clinical practice alongside traditional diagnostic parameters, saliva, in addition to the clinical setting, is more suitable for large-scale population screenings and self-assessment (Guzman et al., 2014).

However, these results require validation in future studies. Commonly used validation techniques, such as enzyme-linked immunosorbent assay (ELISA), Western blot, and protein arrays, have certain limitations, including higher detection limits for some proteins compared to proteomic approaches, the requirement for highly specific antibodies, and an inability to provide a comprehensive analysis of the proteome (Jayasena et al., 2016). Therefore, it may be more appropriate to use SWATH-MS in an independent patient cohort or to employ other proteomic techniques such as SRM (Bostanci et al., 2018).

In conclusion, the periodontal proteome structure in GCF and saliva differs between individuals with periodontitis and those with periodontal health. Differential expression analysis reveals that a considerable proportion of abundant proteins in both sample types are altered in periodontitis, being higher in GCF. Moreover, the most differentially expressed proteins vary between these oral fluids both qualitatively and quantitatively. Therefore, although periodontitis induces changes in the periodontal proteome in GCF and saliva, the expression patterns differ between these fluids.

## Data Availability

The datasets presented in this study can be found in online repositories. The names of the repository/repositories and accession number(s) can be found below: https://www.ebi.ac.uk/pride/archive/, PXD043474 (GCF samples) and PXD043491 (saliva samples).
