# Supplementary material for: Characterisation of the periodontal proteome in gingival crevicular fluid and saliva using SWATH-MS
Source: Front Cell Infect Microbiol. 2025 May 2;15:1576906. doi: 10.3389/fcimb.2025.1576906 (PMC12081464; doi:10.3389/fcimb.2025.1576906)
Supplement: Supplementary file 1 [file Table1.docx]

**Appendix S1.** UniProt accession and protein name of the proteins quantified in periodontal health and periodontitis.

Appendix S1.1. UniProt accession and protein name of the 250 proteins quantified in GCF.

| UniProt accession | Protein name | |  | | UniProt accession | | Protein name | | |
| --- | --- | --- | --- | --- | --- | --- | --- | --- | --- |
| P52209 | 6-phosphogluconate dehydrogenase, decarboxylating | |  | | P31997 | | Carcinoembryonic antigen-related cell adhesion molecule 8 | | |
|  |  |  |  | |  |  |  |  |  |
| P31946 | 14-3-3 protein beta/alpha | |  | | P04040 | | Catalase | | |
| P62258 | 14-3-3 protein epsilon | |  | | P49913 | | Cathelicidin antimicrobial peptide | | |
| P61981 | 14-3-3 protein gamma | |  | | P08311 | | Cathepsin G | | |
| P31947 | 14-3-3 protein sigma | |  | | P29373 | | Cellular retinoic acid-binding protein 2 | | |
| P63104 | 14-3-3 protein zeta/delta | |  | | P00450 | | Ceruloplasmin | | |
| P08865 | 40S ribosomal protein SA | |  | | P36222 | | Chitinase-3-like protein 1 | | |
| P60709 | Actin, cytoplasmic 1 | |  | | O00299 | | Chloride intracellular channel protein 1 | | |
| O15143 | Actin-related protein 2/3 complex subunit 1B | |  | | P10909 | | Clusterin | | |
|  |  |  |  | | P23528 | | Cofilin-1 | | |
| O15144 | Actin-related protein 2/3 complex subunit 2 | |  | | P01024 | | Complement C3 | | |
|  |  |  |  | | P0C0L5 | | Complement C4-B | | |
| P13798 | Acylamino-acid-releasing enzyme | |  | | P00751 | | Complement factor B | | |
| P23526 | Adenosylhomocysteinase | |  | | P08603 | | Complement factor H | | |
| Q01518 | Adenylyl cyclase-associated protein 1 | |  | | Q9UBG3 | | Cornulin | | |
| P43652 | Afamin | |  | | P31146 | | Coronin-1A | | |
| P02768 | Albumin | |  | | P08185 | | Corticosteroid-binding globulin | | |
| P14550 | Aldo-keto reductase family 1 member A1 | |  | | Q9NUQ9 | | CYFIP-related Rac1 interactor B | | |
|  |  |  |  | | P04080 | | Cystatin-B | | |
| P02763 | Alpha-1-acid glycoprotein 1 | |  | | P32320 | | Cytidine deaminase | | |
| P01011 | Alpha-1-antichymotrypsin | |  | | P13716 | | Delta-aminolevulinic acid dehydratase | | |
| P01009 | Alpha-1-antitrypsin | |  | | P26641 | | Elongation factor 1-gamma | | |
| P04217 | Alpha-1B-glycoprotein | |  | | P13639 | | Elongation factor 2 | | |
| P08697 | Alpha-2-antiplasmin | |  | | P11021 | | Endoplasmic reticulum chaperone BiP | | |
| P02765 | Alpha-2-HS-glycoprotein | |  | | P14625 | | Endoplasmin | | |
| P01023 | Alpha-2-macroglobulin | |  | | P12724 | | Eosinophil cationic protein | | |
| A8K2U0 | Alpha-2-macroglobulin-like protein 1 | |  | | P52907 | | F-actin-capping protein subunit alpha-1 | | |
| P12814 | Alpha-actinin-1 | |  | | Q01469 | | Fatty acid-binding protein 5 | | |
| O43707 | Alpha-actinin-4 | |  | | P02671 | | Fibrinogen alpha chain | | |
| P19961 | Alpha-amylase 2B | |  | | P02675 | | Fibrinogen beta chain | | |
| P06733 | Alpha-enolase | |  | | P02679 | | Fibrinogen gamma chain | | |
| P01019 | Angiotensinogen | |  | | P02751 | | Fibronectin | | |
| P04083 | Annexin A1 | |  | | P21333 | | Filamin-A | | |
| P07355 | Annexin A2 | |  | | P04075 | | Fructose-bisphosphate aldolase A | | |
| P12429 | Annexin A3 | |  | | P17931 | | Galectin-3 | | |
| P08133 | Annexin A6 | |  | | P47929 | | Galectin-7 | | |
| P01008 | Antithrombin-III | |  | | Q05315 | | Galectin-10 | | |
| P02647 | Apolipoprotein A-I | |  | | P06744 | | Glucose-6-phosphate isomerase | | |
| P02652 | Apolipoprotein A-II | |  | | P35754 | | Glutaredoxin-1 | | |
| P04114 | Apolipoprotein B-100 | |  | | P00390 | | Glutathione reductase, mitochondrial | | |
| P06576 | ATP synthase subunit beta, mitochondrial | |  | | P78417 | | Glutathione S-transferase omega-1 | | |
|  |  |  |  | | P09211 | | Glutathione S-transferase P | | |
| P20160 | Azurocidin | |  |  | P04406 | | Glyceraldehyde-3-phosphate dehydrogenase | | |
| P02730 | Band 3 anion transport protein | |  | |  |  |  |  |  |
| P02749 | Beta-2-glycoprotein 1 | |  | | P06737 | | Glycogen phosphorylase, liver form | | |
| P17213 | BPI protein | |  |  | P28676 | | Grancalcin | | |
| P04003 | C4b-binding protein alpha chain | |  | | P04899 | | Guanine nucleotide-binding protein G(i) subunit alpha-2 | | |
| P27797 | Calreticulin | |  | |  |  |  |  |  |
| P00915 | Carbonic anhydrase 1 | |  | | P69905 | | Haemoglobin subunit alpha | | |
| P00918 | Carbonic anhydrase 2 | |  | | P68871 | | Haemoglobin subunit beta | | |
| P16152 | Carbonyl reductase [NADPH] 1 | |  | | P02042 | | Haemoglobin subunit delta | | |
| UniProt accession | | Protein name | |  | | UniProt accession | | Protein name |  |
| P69892 | | Haemoglobin subunit gamma-2 | |  | | P08779 | | Keratin, type I cytoskeletal 16 |  |
| P00738 | | Haptoglobin | |  | | Q04695 | | Keratin, type I cytoskeletal 17 |  |
| P00739 | | Haptoglobin-related protein | |  | | P08727 | | Keratin, type I cytoskeletal 19 |  |
| P0DMV9 | | Heat shock 70 kDa protein 1B | |  | | P04264 | | Keratin, type II cytoskeletal 1 |  |
| P11142 | | Heat shock cognate 71 kDa protein | |  | | P35908 | | Keratin, type II cytoskeletal 2 epidermal |  |
| P04792 | | Heat shock protein beta-1 | |  | |  |  |  |  |
| P07900 | | Heat shock protein HSP 90-alpha | |  | | Q01546 | | Keratin, type II cytoskeletal 2 oral |  |
| P02790 | | Hemopexin | |  | | P19013 | | Keratin, type II cytoskeletal 4 |  |
| P05546 | | Heparin cofactor 2 | |  | | P13647 | | Keratin, type II cytoskeletal 5 |  |
| P52790 | | Hexokinase-3 | |  | | P02538 | | Keratin, type II cytoskeletal 6A |  |
| Q9BTM1 | | Histone H2A.J | |  | | P04259 | | Keratin, type II cytoskeletal 6B |  |
| Q99879 | | Histone H2B type 1-M | |  | | P48668 | | Keratin, type II cytoskeletal 6C |  |
| P62805 | | Histone H4 | |  | | P01042 | | Kininogen-1 |  |
| P0DOX2 | | Ig alpha-2 heavy chain | |  | | P02788 | | Lactotransferrin |  |
| P0DOX5 | | Ig gamma-1 heavy chain | |  | | P20700 | | Lamin-B1 |  |
| P01876 | | Ig heavy constant alpha 1 | |  | | P30740 | | Leukocyte elastase inhibitor |  |
| P01859 | | Ig heavy constant gamma 2 | |  | | P09960 | | Leukotriene A-4 hydrolase |  |
| P01860 | | Ig heavy constant gamma 3 | |  | | P00338 | | L-lactate dehydrogenase A chain |  |
| P01871 | | Ig heavy constant mu | |  | | P61626 | | Lysozyme C |  |
| P01742 | | Ig heavy variable 1-69 | |  | | P40121 | | Macrophage-capping protein |  |
| P01764 | | Ig heavy variable 3-23 | |  | | P40925 | | Malate dehydrogenase, cytoplasmic |  |
| P01782 | | Ig heavy variable 3-9 | |  | | P22894 | | Matrix metalloproteinase-8 |  |
| A0A075B6R2 | | Ig heavy variable 4-4 | |  | | P14780 | | Matrix metalloproteinase-9 |  |
| A0A0C4DH38 | | Ig heavy variable 5-51 | |  | | P26038 | | Moesin |  |
| A0A0B4J1U7 | | Ig heavy variable 6-1 | |  | | P24158 | | Myeloblastin |  |
| P01591 | | Ig J chain | |  | | P41218 | | Myeloid cell nuclear differentiation antigen |  |
| P0DOX7 | | Ig kappa light chain | |  | |  |  |  |  |
| P01594 | | Ig kappa variable 1-33 | |  | | P05164 | | Myeloperoxidase |  |
| P04432 | | Ig kappa variable 1D-39 | |  | | P60660 | | Myosin light polypeptide 6 |  |
| A0A087WSZ0 | | Ig kappa variable 1D-8 | |  | | P19105 | | Myosin regulatory light chain 12A |  |
| A0A0C4DH68 | | Ig kappa variable 2-24 | |  | | P35579 | | Myosin-9 |  |
| P01615 | | Ig kappa variable 2D-28 | |  | | P59665 | | Neutrophil defensin 1 |  |
| P04433 | | Ig kappa variable 3-11 | |  | | P08246 | | Neutrophil elastase |  |
| P01624 | | Ig kappa variable 3-15 | |  | | P80188 | | Neutrophil gelatinase-associated lipocalin |  |
| P01619 | | Ig kappa variable 3-20 | |  | |  |  |  |  |
| P06312 | | Ig kappa variable 4-1 | |  | | P43490 | | Nicotinamide phosphoribosyltransferase |  |
| P0DOY3 | | Ig lambda constant 3 | |  | |  |  |  |  |
| P01700 | | Ig lambda variable 1-47 | |  | | P22392 | | Nucleoside diphosphate kinase B |  |
| P01714 | | Ig lambda variable 3-19 | |  | | Q6UX06 | | Olfactomedin-4 |  |
| P80748 | | Ig lambda variable 3-21 | |  | | O75594 | | Peptidoglycan recognition protein 1 |  |
| A0A075B6H9 | | Ig lambda variable 4-69 | |  | | P62937 | | Peptidyl-prolyl cis-trans isomerase A |  |
| P0DOX8 | | Ig lambda-1 light chain | |  | | P62942 | | Peptidyl-prolyl cis-trans isomerase FKBP1A |  |
| P18510 | | IL1ra protein | |  | |  |  |  |  |
| P19827 | | Inter-alpha-trypsin inhibitor heavy chain H1 | |  | | Q06830 | | Peroxiredoxin-1 |  |
|  |  |  |  |  | | P32119 | | Peroxiredoxin-2 |  |
| P19823 | | Inter-alpha-trypsin inhibitor heavy chain H2 | |  | | P30044 | | Peroxiredoxin-5, mitochondrial |  |
|  |  |  |  |  | | P36871 | | Phosphoglucomutase-1 |  |
| Q14624 | | Inter-alpha-trypsin inhibitor heavy chain H4 | |  | | P00558 | | Phosphoglycerate kinase 1 |  |
|  |  |  |  |  | | P18669 | | Phosphoglycerate mutase 1 |  |
| P47756.2 | | Isoform 2 of F-actin-capping protein subunit beta | |  | | Q6P4A8 | | Phospholipase B-like 1 |  |
|  |  |  |  |  | | P05155 | | Plasma protease C1 inhibitor |  |
| P06396.2 | | Isoform 2 of gelsolin | |  | | P00747 | | Plasminogen |  |
| P60174.3 | | Isoform 2 of triosephosphate isomerase | |  | | P13796 | | Plastin-2 |  |
|  |  |  |  |  | | P01833 | | Polymeric Ig receptor |  |
| P35527 | | Keratin, type I cytoskeletal 9 | |  | | P02545 | | Prelamin-A/C |  |
| P13645 | | Keratin, type I cytoskeletal 10 | |  | | P07737 | | Profilin-1 |  |
| P13646 | | Keratin, type I cytoskeletal 13 | |  | | P12273 | | Prolactin-inducible protein |  |
| P02533 | | Keratin, type I cytoskeletal 14 | |  | | O14818 | | Proteasome subunit alpha type-7 |  |
| UniProt accession | Protein name | |  | | UniProt accession | | Protein name | | |
| P02760 | Protein AMBP | |  | | P48595 | | Serpin B10 | | |
| P07237 | Protein disulfide-isomerase | |  | | P02743 | | Serum amyloid P-component | | |
| P30101 | Protein disulfide-isomerase A3 | |  | | Q9H299 | | SH3 domain-binding glutamic acid-rich-like protein 3 | | |
| Q6P5S2 | Protein LEG1 homolog | |  | |  |  |  |  |  |
| P29034 | Protein S100-A2 | |  | | P04179 | | Superoxide dismutase [Mn], mitochondrial | | |
| P06703 | Protein S100-A6 | |  | |  |  |  |  |  |
| P31151 | Protein S100-A7 | |  | | Q99536 | | Synaptic vesicle membrane protein VAT-1 homolog | | |
| P05109 | Protein S100-A8 | |  | |  |  |  |  |  |
| P06702 | Protein S100-A9 | |  | | Q9Y490 | | Talin-1 | | |
| P80511 | Protein S100-A12 | |  | | P10599 | | Thioredoxin | | |
| P25815 | Protein S100-P | |  | | Q9BRA2 | | Thioredoxin domain-containing protein 17 | | |
| Q08188 | Protein-glutamine gamma-glutamyltransferase E | |  | |  |  |  |  |  |
|  |  |  |  | | P19971 | | Thymidine phosphorylase | | |
| Q5VTE0 | Putative elongation factor 1-alpha-like 3 | |  | | P37837 | | Transaldolase | | |
|  |  |  |  | | P55072 | | Transitional endoplasmic reticulum ATPase | | |
| Q5JXB2 | Putative ubiquitin-conjugating enzyme E2 N-like | |  | |  |  |  |  |  |
|  |  |  |  | | P29401 | | Transketolase | | |
| P14618 | Pyruvate kinase PKM | |  |  | P02766 | | Transthyretin | | |
| P50395 | Rab GDP dissociation inhibitor beta | |  | | P67936 | | Tropomyosin alpha-4 chain | | |
| P46940 | Ras GTPase-activating-like protein IQGAP1 | |  | | P62987 | | Ubiquitin-60S ribosomal protein L40 | | |
|  |  |  |  | | P22314 | | Ubiquitin-like modifier-activating enzyme 1 | | |
| P15153 | Ras-related C3 botulinum toxin substrate 2 | |  |  |  |  |  |  |  |
|  |  |  |  | | P08670 | | Vimentin | | |
| P59190 | Ras-related protein Rab-15 | |  | | P18206 | | Vinculin | | |
| Q9HD89 | Resistin | |  | | P02774 | | Vitamin D-binding protein | | |
| P52565 | Rho GDP-dissociation inhibitor 1 | |  | | P04004 | | Vitronectin | | |
| P52566 | Rho GDP-dissociation inhibitor 2 | |  | | O75083 | | WD repeat-containing protein 1 | | |
| P02787 | Serotransferrin | |  |  | P25311 | | Zinc-alpha-2-glycoprotein | | |
| P29508 | Serpin B3 | |  | | Q96DA0 | | Zymogen granule protein 16 homolog B | | |
| P36952 | Serpin B5 | |  | |  | |  | | |

Abbreviations: AMBP: alpha-1-microglobulin/bikunin precursor; ATP: adenosine triphosphate; BiP: binding immunoglobulin protein; BPI: bactericidal permeability-increasing; CYFIP: cytoplasmic Fragile X Messenger Ribonucleoprotein 1-interacting protein; GDP: guanosine diphosphate; GTP: guanosine triphosphate; HS: Heremans Schmid; Ig: immunoglobulin; IL: interleukin; kDa: kilodalton; LEG: liver-enriched gene; Mn: manganese; NADPH: nicotinamide-adenine dinucleotide phosphate; ra: receptor antagonist; Rab: ras-associated binding; Rho: ras homologous; SH: src homology; VAT: vesicle amine transport; WD: Wilson’s disease.

Appendix S1.2. UniProt accession and protein name of the 377 proteins quantified in saliva.

| UniProt accession | Protein name | | |  | | UniProt accession | Protein name | |
| --- | --- | --- | --- | --- | --- | --- | --- | --- |
| Q99943 | 1-acyl-sn-glycerol-3-phosphate acyltransferase alpha | | |  | | P12814 | Alpha-actinin-1 | |
|  |  |  |  |  | | O43707 | Alpha-actinin-4 | |
| P52209 | 6-phosphogluconate dehydrogenase, decarboxylating | | |  | | P0DUB6 | Alpha-amylase 1A | |
|  |  |  |  |  | | P06733 | Alpha-enolase | |
| P61604 | 10 kDa heat shock protein, mitochondrial | | |  | | Q9UBD6 | Ammonium transporter Rh type C | |
|  |  |  |  |  | | P01019 | Angiotensinogen | |
| P31946 | 14-3-3 protein beta/alpha | | |  | | P04083 | Annexin A1 | |
| P62258 | 14-3-3 protein epsilon | | |  | | P07355 | Annexin A2 | |
| P61981 | 14-3-3 protein gamma | | |  | | P12429 | Annexin A3 | |
| P31947 | 14-3-3 protein sigma | | |  | | P08758 | Annexin A5 | |
| P63104 | 14-3-3 protein zeta/delta | | |  | | P08133 | Annexin A6 | |
| P62081 | 40S ribosomal protein S7 | | |  | | Q5VT79 | Annexin A8-like protein 1 | |
| P46783 | 40S ribosomal protein S10 | | |  | | P50995 | Annexin A11 | |
| P62249 | 40S ribosomal protein S16 | | |  | | P03973 | Antileukoproteinase | |
| P08865 | 40S ribosomal protein SA | | |  | | P01008 | Antithrombin-III | |
| P10809 | 60 kDa heat shock protein, mitochondrial | | |  | | P02647 | Apolipoprotein A-I | |
|  |  |  |  |  | | P02652 | Apolipoprotein A-II | |
| P05388 | 60S acidic ribosomal protein P0 | | |  | | P04114 | Apolipoprotein B-100 | |
| P05386 | 60S acidic ribosomal protein P1 | | |  | | P17174 | Aspartate aminotransferase, cytoplasmic | |
| P05387 | 60S acidic ribosomal protein P2 | | |  | |  |  |  |
| Q02878 | 60S ribosomal protein L6 | | |  | | P25705 | ATP synthase subunit alpha, mitochondrial | |
| P30050 | 60S ribosomal protein L12 | | |  | |  |  |  |
| P68032 | Actin, alpha cardiac muscle 1 | | |  | | P06576 | ATP synthase subunit beta, mitochondrial | |
| P60709 | Actin, cytoplasmic 1 | | |  | |  |  |  |
| O15143 | Actin-related protein 2/3 complex subunit 1B | | |  | | P20160 | Azurocidin | |
|  |  |  |  |  | | P02730 | Band 3 anion transport protein | |
| O15144 | Actin-related protein 2/3 complex subunit 2 | | |  | | P02812 | Basic salivary proline-rich protein 2 | |
|  |  |  |  |  | | Q04118 | Basic salivary proline-rich protein 3 | |
| P59998 | Actin-related protein 2/3 complex subunit 4 | | |  | | P02749 | Beta-2-glycoprotein 1 | |
|  |  |  |  |  | | P61769 | Beta-2-microglobulin | |
| P13798 | Acylamino-acid-releasing enzyme | | |  | | Q9NP55 | BPI fold-containing family A member 1 | |
| P23526 | Adenosylhomocysteinase | | |  | | Q96DR5 | BPI fold-containing family A member 2 | |
| Q01518 | Adenylyl cyclase-associated protein 1 | | |  | | Q8TDL5 | BPI fold-containing family B member 1 | |
| P12236 | ADP/ATP translocase 3 | | |  | | Q8N4F0 | BPI fold-containing family B member 2 | |
| P84085 | ADP-ribosylation factor 5 | | |  | | P17213 | BPI protein | |
| P43652 | Afamin | | |  | | P04003 | C4b-binding protein alpha chain | |
| P02768 | Albumin | | |  | | P27482 | Calmodulin-like protein 3 | |
| P30838 | Aldehyde dehydrogenase, dimeric NADP-preferring | | |  | | P07384 | Calpain-1 catalytic subunit | |
|  |  |  |  |  | | P27797 | Calreticulin | |
| P14550 | Aldo-keto reductase family 1 member A1 | | |  | | P00915 | Carbonic anhydrase 1 | |
|  |  |  |  |  | | P00918 | Carbonic anhydrase 2 | |
| O60218 | Aldo-keto reductase family 1 member B10 | | |  | | P23280 | Carbonic anhydrase 6 | |
|  |  |  |  |  | | P16152 | Carbonyl reductase [NADPH] 1 | |
| P02763 | Alpha-1-acid glycoprotein 1 | | |  | | P13688 | Carcinoembryonic antigen-related cell adhesion molecule 1 | |
| P19652 | Alpha-1-acid glycoprotein 2 | | |  | |  |  |  |
| P01011 | Alpha-1-antichymotrypsin | | |  | | P04040 | Catalase | |
| P01009 | Alpha-1-antitrypsin | | |  | | P49913 | Cathelicidin antimicrobial peptide | |
| P04217 | Alpha-1B-glycoprotein | | |  | | P07339 | Cathepsin D | |
| P08697 | Alpha-2-antiplasmin | | |  | | P08311 | Cathepsin G | |
| P02765 | Alpha-2-HS-glycoprotein | | |  | | P60953 | Cell division control protein 42 homolog | |
| P01023 | Alpha-2-macroglobulin | | |  | | P29373 | Cellular retinoic acid-binding protein 2 | |
| A8K2U0 | Alpha-2-macroglobulin-like protein 1 | | |  | | P00450 | Ceruloplasmin | |
|  |  | | |  | |  |  | |
|  |  | | |  | |  |  | |
| UniProt accession | Protein name | |  | | UniProt accession | | | Protein name |
| P36222 | Chitinase-3-like protein 1 | |  | | P04075 | | | Fructose-bisphosphate aldolase A |
| Q13231 | Chitotriosidase-1 | |  | | P17931 | | | Galectin-3 |
| O00299 | Chloride intracellular channel protein 1 | |  | | Q08380 | | | Galectin-3-binding protein |
|  |  |  |  | | P47929 | | | Galectin-7 |
| O95833 | Chloride intracellular channel protein 3 | |  | | P06396 | | | Gelsolin |
|  |  |  |  | | P06744 | | | Glucose-6-phosphate isomerase |
| Q00610 | Clathrin heavy chain 1 | |  | | P00390 | | | Glutathione reductase, mitochondrial |
| P10909 | Clusterin | |  | | P78417 | | | Glutathione S-transferase omega-1 |
| O00748 | Cocaine esterase | |  | | P09211 | | | Glutathione S-transferase P |
| P23528 | Cofilin-1 | |  | | P04406 | | | Glyceraldehyde-3-phosphate dehydrogenase |
| P01024 | Complement C3 | |  | |  |  |  |  |
| P0C0L4 | Complement C4-A | |  | | P06737 | | | Glycogen phosphorylase, liver form |
| P00751 | Complement factor B | |  | | P28676 | | | Grancalcin |
| P08603 | Complement factor H | |  | | P62826 | | | GTP-binding nuclear protein Ran |
| Q9UBG3 | Cornulin | |  | | P04899 | | | Guanine nucleotide-binding protein G(i) subunit alpha-2 |
| P31146 | Coronin-1A | |  | |  |  |  |  |
| P08185 | Corticosteroid-binding globulin | |  | | Q6ZN66 | | | Guanylate-binding protein 6 |
| Q9NUQ9 | CYFIP-related Rac1 interactor B | |  | | P69905 | | | Haemoglobin subunit alpha |
| P01040 | Cystatin-A | |  | | P68871 | | | Haemoglobin subunit beta |
| P04080 | Cystatin-B | |  | | P02042 | | | Haemoglobin subunit delta |
| P01034 | Cystatin-C | |  | | P69892 | | | Haemoglobin subunit gamma-2 |
| P28325 | Cystatin-D | |  | | P00738 | | | Haptoglobin |
| P01036 | Cystatin-S | |  | | P00739 | | | Haptoglobin-related protein |
| P09228 | Cystatin-SA | |  | | P0DMV9 | | | Heat shock 70 kDa protein 1B |
| P01037 | Cystatin-SN | |  | | P11142 | | | Heat shock cognate 71 kDa protein |
| P54108 | Cysteine-rich secretory protein 3 | |  | | P04792 | | | Heat shock protein beta-1 |
| P32320 | Cytidine deaminase | |  | | P08238 | | | Heat shock protein HSP 90-beta |
| P04839 | Cytochrome b-245 heavy chain | |  | | P02790 | | | Hemopexin |
| P13716 | Delta-aminolevulinic acid dehydratase | |  | | P05546 | | | Heparin cofactor 2 |
| P32926 | Desmoglein-3 | |  | | O60506 | | | Heterogeneous nuclear ribonucleoprotein Q |
| P15924 | Desmoplakin | |  | |  |  |  |  |
| P36957 | Dihydrolipoyllysine-residue succinyltransferase component of 2-oxoglutarate dehydrogenase complex, mitochondrial | |  | | P52790 | | | Hexokinase-3 |
|  |  |  |  | | P04196 | | | Histidine-rich glycoprotein |
|  |  |  |  | | P07305 | | | Histone H1.0 |
|  |  |  |  | | P16402 | | | Histone H1.3 |
| P26641 | Elongation factor 1-gamma | |  | | P16401 | | | Histone H1.5 |
| P13639 | Elongation factor 2 | |  | | Q93077 | | | Histone H2A type 1-C |
| Q9NZ08 | Endoplasmic reticulum aminopeptidase 1 | |  | | Q9BTM1 | | | Histone H2A.J |
|  |  |  |  | | Q99879 | | | Histone H2B type 1-M |
| P11021 | Endoplasmic reticulum chaperone BiP | |  | | P68431 | | | Histone H3.1 |
| P14625 | Endoplasmin | |  | | P62805 | | | Histone H4 |
| Q92817 | Envoplakin | |  | | P0DOX2 | | | Ig alpha-2 heavy chain |
| P12724 | Eosinophil cationic protein | |  | | P0DOX3 | | | Ig delta heavy chain |
| Q96HE7 | ERO1-like protein alpha | |  | | P0DOX5 | | | Ig gamma-1 heavy chain |
| P60842 | Eukaryotic initiation factor 4A-I | |  | | P01876 | | | Ig heavy constant alpha 1 |
| Q6IS14 | Eukaryotic translation initiation factor 5A-1-like | |  | | P01859 | | | Ig heavy constant gamma 2 |
|  |  |  |  | | P01860 | | | Ig heavy constant gamma 3 |
| Q16610 | Extracellular matrix protein 1 | |  | | P01861 | | | Ig heavy constant gamma 4 |
| P52907 | F-actin-capping protein subunit alpha-1 | |  | | P01871 | | | Ig heavy constant mu |
|  |  |  |  | | P01742 | | | Ig heavy variable 1-69 |
| Q01469 | Fatty acid-binding protein 5 | |  | | P01764 | | | Ig heavy variable 3-23 |
| Q6ZVX7 | F-box only protein 50 | |  | | A0A0B4J1V6 | | | Ig heavy variable 3-73 |
| P02671 | Fibrinogen alpha chain | |  | | P01782 | | | Ig heavy variable 3-9 |
| P02675 | Fibrinogen beta chain | |  | | A0A075B6R2 | | | Ig heavy variable 4-4 |
| P02679 | Fibrinogen gamma chain | |  | | A0A0C4DH38 | | | Ig heavy variable 5-51 |
| P02751 | Fibronectin | |  | | P01591 | | | Ig J chain |
| Q5D862 | Filaggrin-2 | |  | | P0DOX7 | | | Ig kappa light chain |
| P21333 | Filamin-A | |  | | P01594 | | | Ig kappa variable 1-33 |
| UniProt accession | | Protein name | |  | | UniProt accession | Protein name | |
| P04432 | | Ig kappa variable 1D-39 | |  | | P01042 | Kininogen-1 | |
| A0A087WSZ0 | | Ig kappa variable 1D-8 | |  | | P22079 | Lactoperoxidase | |
| A0A0C4DH68 | | Ig kappa variable 2-24 | |  | | P02788 | Lactotransferrin | |
| P01615 | | Ig kappa variable 2D-28 | |  | | P20700 | Lamin-B1 | |
| P04433 | | Ig kappa variable 3-11 | |  | | P55268 | Laminin subunit beta-2 | |
| P01624 | | Ig kappa variable 3-15 | |  | | P30740 | Leukocyte elastase inhibitor | |
| P01619 | | Ig kappa variable 3-20 | |  | | P09960 | Leukotriene A-4 hydrolase | |
| P06312 | | Ig kappa variable 4-1 | |  | | P31025 | Lipocalin-1 | |
| P0DOY3 | | Ig lambda constant 3 | |  | | P00338 | L-lactate dehydrogenase A chain | |
| P01700 | | Ig lambda variable 1-47 | |  | | P07195 | L-lactate dehydrogenase B chain | |
| P01714 | | Ig lambda variable 3-19 | |  | | P61626 | Lysozyme C | |
| P80748 | | Ig lambda variable 3-21 | |  | | P14174 | Macrophage migration inhibitory factor | |
| P01717 | | Ig lambda variable 3-25 | |  | | P40121 | Macrophage-capping protein | |
| P0DOX8 | | Ig lambda-1 light chain | |  | | P40925 | Malate dehydrogenase, cytoplasmic | |
| Q9Y6R7 | | IgGFc-binding protein | |  | | P40926 | Malate dehydrogenase, mitochondrial | |
| P18510 | | IL1ra protein | |  | | P22894 | Matrix metalloproteinase-8 | |
| P11215 | | Integrin alpha-M | |  | | P14780 | Matrix metalloproteinase-9 | |
| P19827 | | Inter-alpha-trypsin inhibitor heavy chain H1 | |  | | P01033 | Metalloproteinase inhibitor 1 | |
|  |  |  |  |  | | P26038 | Moesin | |
| P19823 | | Inter-alpha-trypsin inhibitor heavy chain H2 | |  |  | Q9HC84 | Mucin-5B | |
|  |  |  |  |  | | Q8TAX7 | Mucin-7 | |
| Q14624 | | Inter-alpha-trypsin inhibitor heavy chain H4 | |  | | P24158 | Myeloblastin | |
|  |  |  |  |  | | P41218 | Myeloid cell nuclear differentiation antigen | |
| P23280.2 | | Isoform 2 of carbonic anhydrase 6 | |  |  |  |  |  |
| Q02487.2 | | Isoform 2 of desmocollin-2 | |  | | P05164 | Myeloperoxidase | |
| P47756.2 | | Isoform 2 of F-actin-capping protein subunit beta | |  | | P60660 | Myosin light polypeptide 6 | |
|  |  |  |  |  | | P19105 | Myosin regulatory light chain 12A | |
| P60174.3 | | Isoform 2 of triosephosphate isomerase | |  | | P35579 | Myosin-9 | |
|  |  |  |  |  |  | Q9UJ70 | N-acetyl-D-glucosamine kinase | |
| P06753.2 | | Isoform 2 of tropomyosin alpha-3 chain | |  | | Q09666 | Neuroblast differentiation-associated protein AHNAK | |
| Q9UGM3.8 | | Isoform 8 of deleted in malignant brain tumours 1 protein | |  |  | Q14697 | Neutral alpha-glucosidase AB | |
|  |  |  |  |  | | P59665 | Neutrophil defensin 1 | |
| P11413.2 | | Isoform long of glucose-6-phosphate 1-dehydrogenase | |  | | P08246 | Neutrophil elastase | |
|  |  |  |  |  | | P80188 | Neutrophil gelatinase-associated lipocalin | |
| P14923 | | Junction plakoglobin | |  | |  |  |  |
| P06870 | | Kallikrein-1 | |  | | P43490 | Nicotinamide phosphoribosyltransferase | |
| O76013 | | Keratin, type I cuticular Ha6 | |  | |  |  |  |
| P35527 | | Keratin, type I cytoskeletal 9 | |  | | P80303 | Nucleobindin-2 | |
| P13645 | | Keratin, type I cytoskeletal 10 | |  | | P22392 | Nucleoside diphosphate kinase B | |
| P13646 | | Keratin, type I cytoskeletal 13 | |  | | Q6UX06 | Olfactomedin-4 | |
| P02533 | | Keratin, type I cytoskeletal 14 | |  | | Q96FX8 | p53 apoptosis effector related to PMP-22 | |
| P19012 | | Keratin, type I cytoskeletal 15 | |  | |  |  |  |
| P08779 | | Keratin, type I cytoskeletal 16 | |  | | O75594 | Peptidoglycan recognition protein 1 | |
| Q04695 | | Keratin, type I cytoskeletal 17 | |  | | P62937 | Peptidyl-prolyl cis-trans isomerase A | |
| P08727 | | Keratin, type I cytoskeletal 19 | |  | | P23284 | Peptidyl-prolyl cis-trans isomerase B | |
| Q9NSB2 | | Keratin, type II cuticular Hb4 | |  | | P62942 | Peptidyl-prolyl cis-trans isomerase FKBP1A | |
| P04264 | | Keratin, type II cytoskeletal 1 | |  | |  |  |  |
| P35908 | | Keratin, type II cytoskeletal 2 epidermal | |  | | O60437 | Periplakin | |
|  |  |  |  |  | | Q06830 | Peroxiredoxin-1 | |
| Q01546 | | Keratin, type II cytoskeletal 2 oral | |  | | P32119 | Peroxiredoxin-2 | |
| P12035 | | Keratin, type II cytoskeletal 3 | |  | | P30044 | Peroxiredoxin-5, mitochondrial | |
| P19013 | | Keratin, type II cytoskeletal 4 | |  | | P30041 | Peroxiredoxin-6 | |
| P13647 | | Keratin, type II cytoskeletal 5 | |  | | P30086 | Phosphatidylethanolamine-binding protein 1 | |
| P02538 | | Keratin, type II cytoskeletal 6A | |  | |  |  |  |
| P04259 | | Keratin, type II cytoskeletal 6B | |  | | P36871 | Phosphoglucomutase-1 | |
| P48668 | | Keratin, type II cytoskeletal 6C | |  | | P00558 | Phosphoglycerate kinase 1 | |
| Q8N1N4 | | Keratin, type II cytoskeletal 78 | |  | | P18669 | Phosphoglycerate mutase 1 | |
| UniProt accession | Protein name | | |  | | UniProt accession | Protein name | |
| Q6P4A8 | Phospholipase B-like 1 | | |  | | P61026 | Ras-related protein Rab-10 | |
| Q13835 | Plakophilin-1 | | |  | | Q9HD89 | Resistin | |
| Q9Y446 | Plakophilin-3 | | |  | | P52565 | Rho GDP-dissociation inhibitor 1 | |
| P05155 | Plasma protease C1 inhibitor | | |  | | P52566 | Rho GDP-dissociation inhibitor 2 | |
| P00747 | Plasminogen | | |  | | Q07960 | Rho GTPase-activating protein 1 | |
| P13796 | Plastin-2 | | |  | | Q96QR1 | Secretoglobin family 3A member 1 | |
| P13797 | Plastin-3 | | |  | | P02787 | Serotransferrin | |
| P01833 | Polymeric Ig receptor | | |  | | P29508 | Serpin B3 | |
| P02545 | Prelamin-A/C | | |  | | P36952 | Serpin B5 | |
| P07737 | Profilin-1 | | |  | | P48595 | Serpin B10 | |
| O75340 | Programmed cell death protein 6 | | |  | | Q9UIV8 | Serpin B13 | |
| P12273 | Prolactin-inducible protein | | |  | | P02743 | Serum amyloid P-component | |
| Q16651 | Prostasin | | |  | | Q9H299 | SH3 domain-binding glutamic acid-rich-like protein 3 | |
| P60900 | Proteasome subunit alpha type-6 | | |  | |  |  |  |
| O14818 | Proteasome subunit alpha type-7 | | |  | | Q9UBC9 | Small proline-rich protein 3 | |
| P02760 | Protein AMBP | | |  | | Q14515 | SPARC-like protein 1 | |
| P07237 | Protein disulfide-isomerase | | |  | | O00391 | Sulfhydryl oxidase 1 | |
| P30101 | Protein disulfide-isomerase A3 | | |  | | P04179 | Superoxide dismutase [Mn], mitochondrial | |
| Q6P5S2 | Protein LEG1 homolog | | |  | |  |  |  |
| Q96TA1 | Protein Niban 2 | | |  | | Q99536 | Synaptic vesicle membrane protein VAT-1 homolog | |
| Q8WVV4 | Protein POF1B | | |  | |  |  |  |
| P29034 | Protein S100-A2 | | |  | | Q9Y490 | Talin-1 | |
| P06703 | Protein S100-A6 | | |  | | P10599 | Thioredoxin | |
| P31151 | Protein S100-A7 | | |  | | P19971 | Thymidine phosphorylase | |
| P05109 | Protein S100-A8 | | |  | | P37837 | Transaldolase | |
| P06702 | Protein S100-A9 | | |  | | P20061 | Transcobalamin-1 | |
| P31949 | Protein S100-A11 | | |  | | P37802 | Transgelin-2 | |
| P80511 | Protein S100-A12 | | |  | | P55072 | Transitional endoplasmic reticulum ATPase | |
| Q9HCY8 | Protein S100-A14 | | |  | |  |  |  |
| Q96FQ6 | Protein S100-A16 | | |  | | P29401 | Transketolase | |
| P25815 | Protein S100-P | | |  | | Q86T26 | Transmembrane protease serine 11B | |
| Q08188 | Protein-glutamine gamma-glutamyltransferase E | | |  | | P02766 | Transthyretin | |
|  |  |  |  |  | | Q9BQE3 | Tubulin alpha-1C chain | |
| P22735 | Protein-glutamine gamma-glutamyltransferase K | | |  | | P68366 | Tubulin alpha-4A chain | |
|  |  |  |  |  | | P68371 | Tubulin beta-4B chain | |
| P00491 | Purine nucleoside phosphorylase | | |  | | P62987 | Ubiquitin-60S ribosomal protein L40 | |
| P55786 | Puromycin-sensitive aminopeptidase | | |  | | P22314 | Ubiquitin-like modifier-activating enzyme 1 | |
| Q5VTE0 | Putative elongation factor 1-alpha-like 3 | | |  | |  |  |  |
|  |  |  |  |  | | P08670 | Vimentin | |
| Q5JXB2 | Putative ubiquitin-conjugating enzyme E2 N-like | | |  | | P18206 | Vinculin | |
|  |  |  |  |  | | P02774 | Vitamin D-binding protein | |
| P14618 | Pyruvate kinase PKM | | |  | | P04004 | Vitronectin | |
| P50395 | Rab GDP dissociation inhibitor beta | | |  | | P21796 | Voltage-dependent anion-selective channel protein 1 | |
| P46940 | Ras GTPase-activating-like protein IQGAP1 | | |  | |  |  |  |
|  |  |  |  |  | | O75083 | WD repeat-containing protein 1 | |
| P15153 | Ras-related C3 botulinum toxin substrate 2 | | |  | | P25311 | Zinc-alpha-2-glycoprotein | |
|  |  |  |  |  | | Q96DA0 | Zymogen granule protein 16 homolog B | |

Abbreviations: ADP: adenosine diphosphate; AMBP: alpha-1-microglobulin/bikunin precursor; ATP: adenosine triphosphate; BiP: binding immunoglobulin protein; BPI: bactericidal permeability-increasing; CYFIP: cytoplasmic Fragile X Messenger Ribonucleoprotein 1-interacting protein; ERO: endoplasmic reticulum oxidoreductin; GDP: guanosine diphosphate; GTP: guanosine triphosphate; HS: Heremans Schmid; Ig: immunoglobulin; IgGFc: immunoglobulin G fragment crystallisable; IL: interleukin; kDa: kilodalton; LEG: liver-enriched gene; Mn: manganese; NADPH: nicotinamide-adenine dinucleotide phosphate; PMP: peripheral myelin protein; POF: premature ovarian failure; ra: receptor antagonist; Rab: ras-associated binding; Rh: rhesus; Rho: ras homologous; SH: src homology; SPARC: secreted protein acidic and rich in cysteine; VAT: vesicle amine transport; WD: Wilson’s disease.
