# Supplementary material for: Characterisation of the periodontal proteome in gingival crevicular fluid and saliva using SWATH-MS
Source: Front Cell Infect Microbiol. 2025 May 2;15:1576906. doi: 10.3389/fcimb.2025.1576906 (PMC12081464; doi:10.3389/fcimb.2025.1576906)

**Appendix S2.** Eigenvalue of the percentage of variability explained within each dimension in the GCF and saliva samples.

Appendix S2.1. Eigenvalue of the percentage of variability explained within each dimension in the GCF samples.


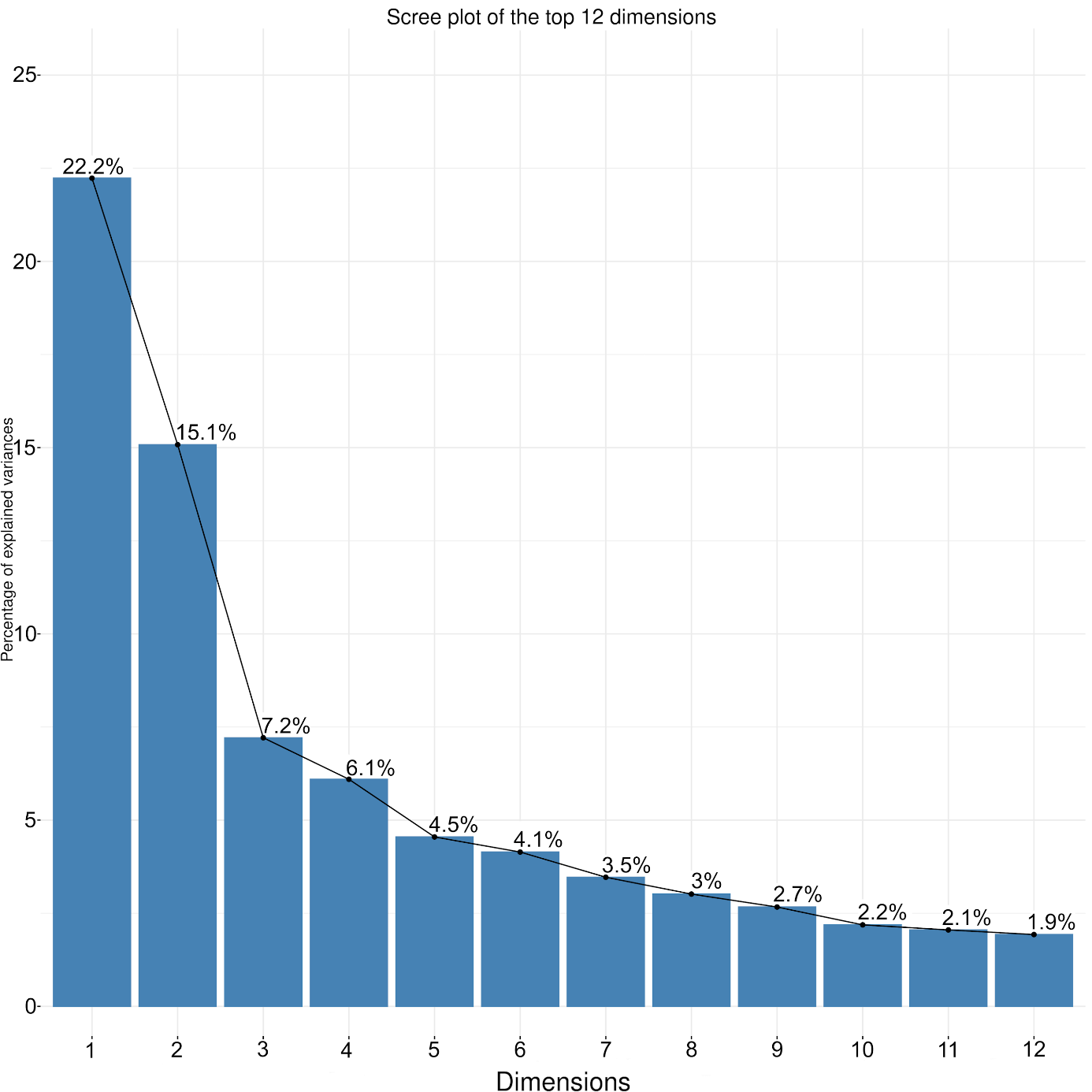


Appendix S2.2. Eigenvalue of the percentage of variability explained within each dimension in the saliva samples.


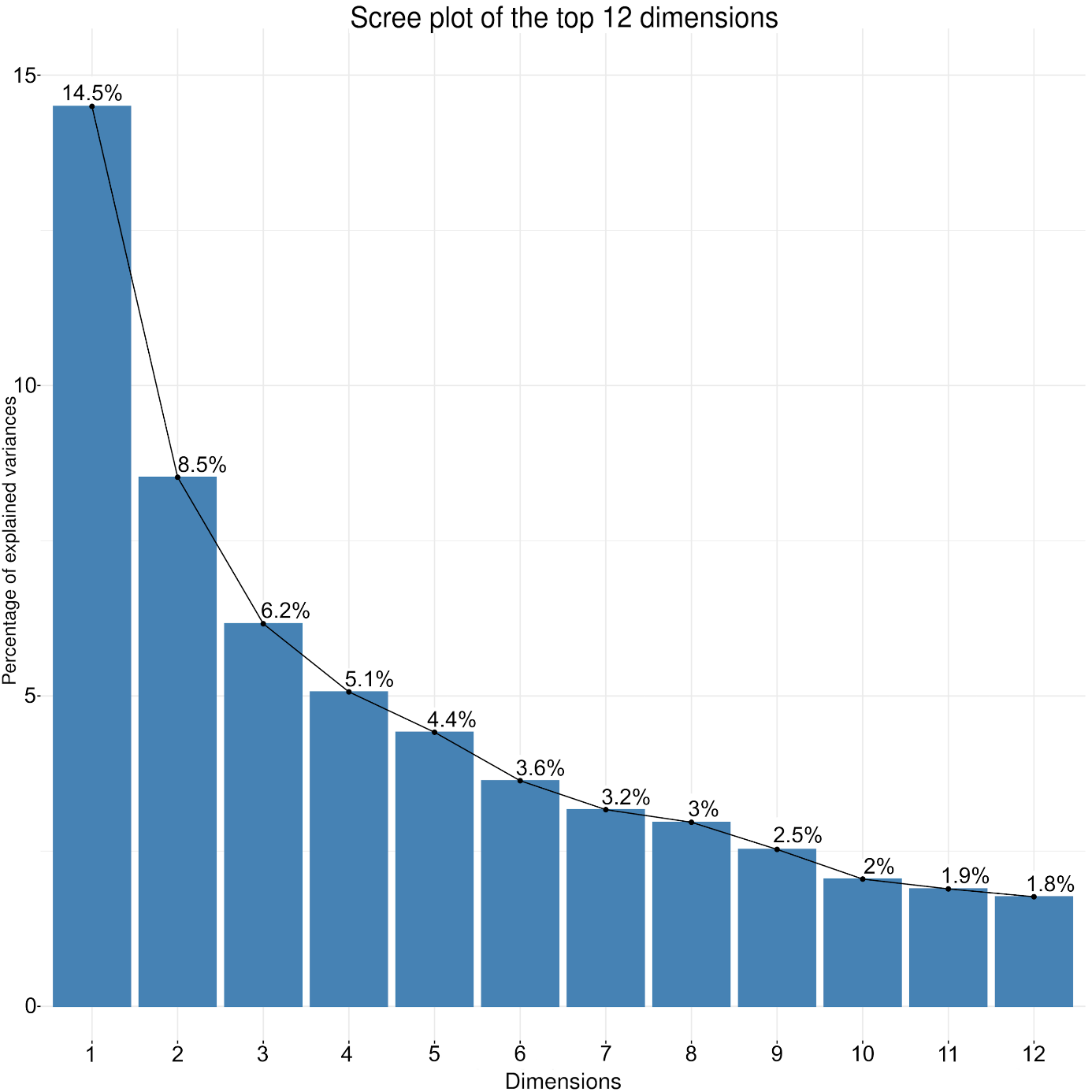

Supplement: Supplementary file 2 [file Table2.docx]
